# Whole-Genome Sequence Data Suggest Environmental Adaptation of Ethiopian Sheep Populations

**DOI:** 10.1093/gbe/evab014

**Published:** 2021-01-27

**Authors:** Pamela Wiener, Christelle Robert, Abulgasim Ahbara, Mazdak Salavati, Ayele Abebe, Adebabay Kebede, David Wragg, Juliane Friedrich, Deepali Vasoya, David A Hume, Appolinaire Djikeng, Mick Watson, James G D Prendergast, Olivier Hanotte, Joram M Mwacharo, Emily L Clark

**Affiliations:** 1 Roslin Institute, University of Edinburgh, Midlothian, United Kingdom; 2 Centre for Tropical Livestock Genetics and Health (CTLGH), Midlothian, United Kingdom; 3 School of Life Sciences, University of Nottingham, United Kingdom; 4 Department of Zoology, Misurata University, Misurata, Libya; 5 Debre Berhan Research Centre, Debre Berhan, Ethiopia; 6 Amhara Regional Agricultural Research Institute, Bahir Dar, Ethiopia; 7 LiveGene, International Livestock Research Institute, Addis Ababa, Ethiopia; 8 Mater Research Institute-University of Queensland, Translational Research Institute, Brisbane, Australia; 9 Animal and Veterinary Sciences Group, Scotland’s Rural College (SRUC), Midlothian, United Kingdom; 10 Small Ruminant Genomics, International Centre for Agricultural Research in the Dry Areas (ICARDA), Addis Ababa, Ethiopia

**Keywords:** selection, precipitation, ruminants

## Abstract

Great progress has been made over recent years in the identification of selection signatures in the genomes of livestock species. This work has primarily been carried out in commercial breeds for which the dominant selection pressures are associated with artificial selection. As agriculture and food security are likely to be strongly affected by climate change, a better understanding of environment-imposed selection on agricultural species is warranted. Ethiopia is an ideal setting to investigate environmental adaptation in livestock due to its wide variation in geo-climatic characteristics and the extensive genetic and phenotypic variation of its livestock. Here, we identified over three million single nucleotide variants across 12 Ethiopian sheep populations and applied landscape genomics approaches to investigate the association between these variants and environmental variables. Our results suggest that environmental adaptation for precipitation-related variables is stronger than that related to altitude or temperature, consistent with large-scale meta-analyses of selection pressure across species. The set of genes showing association with environmental variables was enriched for genes highly expressed in human blood and nerve tissues. There was also evidence of enrichment for genes associated with high-altitude adaptation although no strong association was identified with hypoxia-inducible-factor (HIF) genes. One of the strongest altitude-related signals was for a collagen gene, consistent with previous studies of high-altitude adaptation. Several altitude-associated genes also showed evidence of adaptation with temperature, suggesting a relationship between responses to these environmental factors. These results provide a foundation to investigate further the effects of climatic variables on small ruminant populations.

**Significance**

A better understanding of environmental adaptation in native livestock breeds will inform breeding and management strategies and may aid in facilitating greater production efficiency without excessive reliance on external inputs. This study exploited the genetic and phenotypic diversity of livestock from a single country (Ethiopia), with extensive variation in agro-ecological characteristics, to investigate environmental adaptation. This experimental approach is likely to reduce the detection of false positive results that can arise in selection scans due to the role of nonselective demographic factors. Based on the analysis of ∼100 local sheep genomes collected across different agro-ecological zones in Ethiopia, this study found stronger evidence of adaptation driven by precipitation levels compared to that associated with temperature or altitude, suggesting that rainfall is an important selective pressure on Ethiopian sheep.

## Introduction

Over the last 20 years, livestock geneticists have exploited the development of genomic tools and resources to identify regions of the genome showing evidence of selection. The design of these experiments and the choice of breeds has resulted in a focus on identification of signatures primarily related to artificial selection in commercial breeds (Gutierrez-Gil et al. 2015; Randhawa et al. 2016). However, over the same period, concern has been rising regarding the effects of climate change on agriculture, indicating that a better understanding of natural selection on livestock genomes is necessary. As genomic signatures of human-imposed selection tend to dominate over those from environmental selection in high-production breeds, focusing on native breeds may be more informative in this regard (Biscarini et al. 2015).

Characterizing environmental adaptation in plants and animals has traditionally been an expensive and time-consuming endeavor, requiring large-scale experiments carried out over a range of environmental conditions. Such experiments are generally prohibitively expensive and challenging to carry out for non-model organisms with long generation times. However, the development of global climate data sets (Fick and Hijmans 2017) now provide detailed climatic information from across the world, allowing an indirect approach for studying genetic adaptation to environmental factors. Thus, locations where organisms are found can be used as proxies for environmental adaptation phenotypes. This “landscape genomics” approach (Manel et al. 2003) was initially mainly applied to wild species but can also be applied to livestock (Bertolini et al. 2018; Flori et al. 2019; Joost et al. 2008), with the additional complication that the location of particular livestock genotypes is influenced by both natural and human-directed forces.

To apply such an approach to the study of environmental adaptation in livestock, the ideal experimental design would involve sampling of animals adapted to a range of climatic conditions. Ethiopia is characterized by a range of related environmental conditions, varying widely in rainfall (long-term average annual levels: <500–2000 mm; Gebrechorkos et al. 2019) and altitude (−125 to 4550 m; Central Intelligence Agency 2021) and also varying in daily and seasonal temperature (Gebrechorkos et al. 2019). Seasonal rainfall in Ethiopia is influenced by large-scale climatic variability, including the El Niño Southern Oscillation (ENS), Indian Ocean Dipole (IOD), and migration of the Inter‐Tropical Convergence Zone (ITCZ), leading to changes in the timing and length of the rainy season(s) between years and resulting in frequent droughts and flooding (Gebrechorkos et al. 2019; McSweeney et al. 2010a, 2010b).

Ethiopia makes up the greater part of the “Horn of Africa,” which is thought to have been a gateway of introduction of both cattle and sheep from the Middle East into Africa, resulting in extensive genetic diversity of native livestock (Edea et al. 2017; Muigai and Hanotte 2013). Sheep are distributed across most eco-environments in the country and there is marked genetic and phenotypic differentiation between populations (Ahbara et al. 2019; Edea et al. 2019; Gizaw et al. 2007). Currently, the Ethiopian livestock sector constitutes a substantial component of the economy and sustains most family farms (FAO 2014, 2018). While cattle make up the largest part of the sector, sheep production is also an important component of livestock farming; approximately a third of smallholder farmers own sheep (Negassa and Jabbar 2008) and they provide a range of products (meat, milk, skin, manure). Sheep have been the target of recent community-based breeding strategies (Haile et al. 2013; Recha et al. 2019) due to their affordability to subsistence farmers, their tendency to be cared for by women and children and their greater adaptation to marginal environments.

This study exploits the existing distribution of Ethiopian indigenous sheep across a range of agro-ecological and climatic conditions to examine the genetic basis of environmental adaption. By performing whole-genome sequencing (WGS), we generated dense genotypic data for 12 populations across the country. We then used landscape genomic approaches to examine associations between genetic markers and environmental measures. This allowed us to determine which measures potentially show the strongest evidence of environmental selective pressure on the sheep genome and associated candidate genes.

## Results

**Fig. 1:**
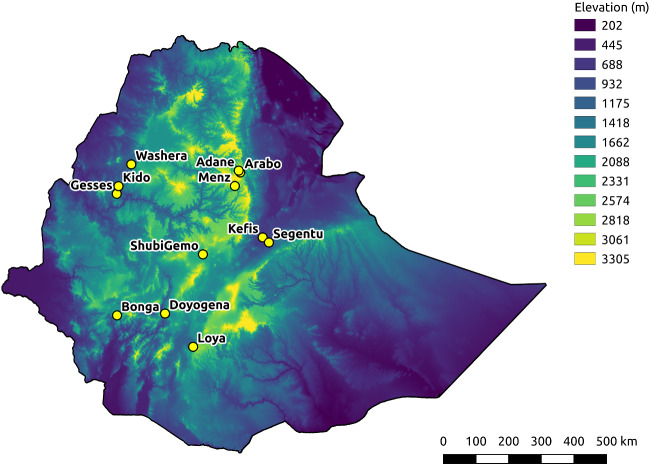
Altitude-coded map of Ethiopia with 12 sampling locations indicated.

To investigate environmental adaptation between sheep populations, we analyzed WGS data, at a mean coverage of 54X, for 94 animals across 12 Ethiopian locations and environmental conditions (table 1, fig. 1).

**Table 1:** Description of Ethiopian Sheep Populations

Population	CODE	Post-QC sample size	Tail phenotype	Horn phenotype	Latitude	Longitude	Altitude (m)	Average SNP diversity
Adane	AKD	8	Fat-rump	Polled	11.1416	39.5	2,783	0.32
Arabo	AKR	8	Fat-rump	Polled	11.0904	39.5425	2,610	0.32
Bonga	BO	10	Long fat-tailed	Polled	7.16	36.15	1,949	0.28
Doyogena	DA	10	Long fat-tailed	Short and straight horn	7.211	37.4711	1,348	0.30
Kefis	FKD	8	Fat-rump	Polled	9.3044	40.16	740	0.32
Segentu	FSG	4	Fat-rump	Polled	9.1645	40.332	859	0.32
Gesses	GGD	6	Long fat-tailed	Polled	10.5032	36.1412	1,658	0.30
Kido	KO	5	Long fat-tailed	Polled	10.714	36.191	1,315	0.30
Loya	LA	8	Long fat-tailed	Short horned, curved upward	6.2947	38.2451	1,911	0.29
Menz^a^	MZ	10	Short fat-tailed	Twisted horn	10.718	39.3939	2,668	0.31
Shubi Gemo	SHG	8	Long fat-tailed	Short and straight horn	8.838	38.5142	2,067	0.31
Washera^b^	WA	9	Short fat-tailed	Polled	11.3133	36.5422	1,177	0.30

a Referred to as Molale-Menz in Ahbara et al. (2019).

b Referred to as Gafera-Washera in Ahbara et al. (2019).

### Relationships Between Sheep Populations

**Fig. 2:**
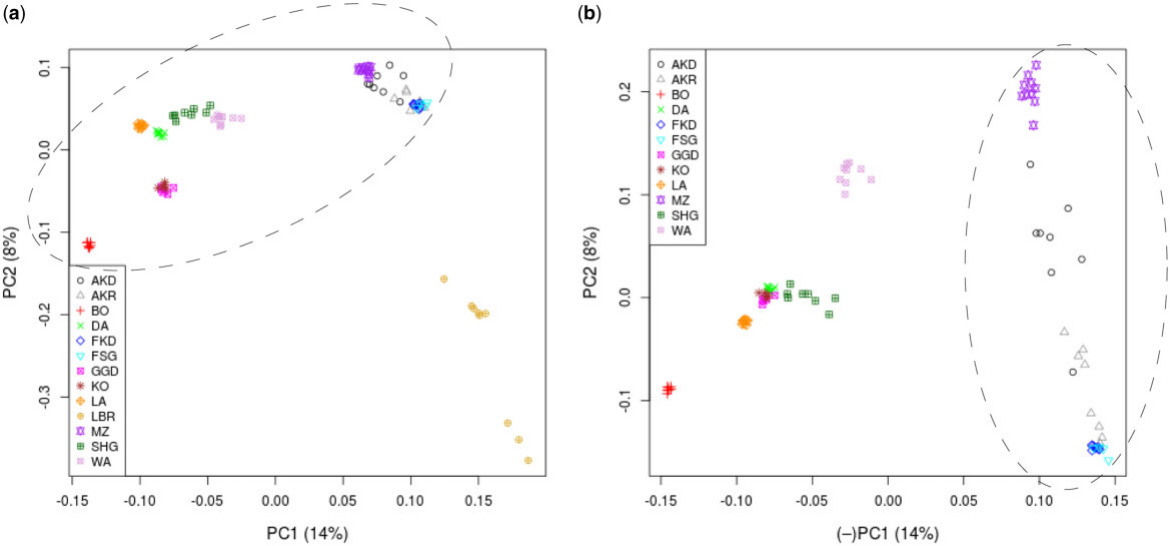
PC1 versus PC2 for PCA of genomic data: (*a*) Ethiopian (ringed) and Libyan (LBR) sheep populations. (*b*) Only Ethiopian populations (most eastern populations, used for PBS analysis, are ringed).

A previous study characterized the genetic relationships between the Ethiopian sheep populations (with the exception of one population, Segentu) and two Sudanese breeds (not included in the current study), based on data from the Ovine 50 K SNP BeadChip (Ahbara et al. 2019). Analysis of genetic structure by Admixture and PCA revealed genetic differentiation between populations; the Admixture results supported four genetic clusters (one of which was strongly associated with the Sudanese breeds). The structure was related to geographical region and, to some extent, tail phenotypes (i.e., long or short fat tail and fat rump) of the populations. To further investigate the genetic relationships between the Ethiopian populations, we performed PCA based on the WGS data, both with and without a Libyan population (LBR), treated as an outgroup (fig. 2*a* and *b*). The pattern was very similar to that derived from the Ahbara et al. (2019) study. Again, the populations were separated geographically, with PC1 associated with the west-east gradient, such that the right-hand-side of the plots are associated with more easterly locations. The Segentu population (new to this study) clustered tightly with the geographically nearby Kefis population, within the group of more eastern Ethiopian populations (Adane, Arabo, Menz, Kefis, identified as Cluster C by Ahbara et al. (2019)), which is composed of both high- and low-altitude populations. This group of populations (with the exception of Kefis and Segentu) showed differentiation along the PC2 axis of the PCA with only the Ethiopian populations (fig. 2*b*). In addition, as for Kefis and Segentu, the Gesses and Kido populations were not separated by either PCA (with and without LBR). For both analyses, the first two principal components accounted for 14% and 8% of the variation, respectively. We also calculated pairwise F_ST_ for the 13 populations (supplementary table S1, supplementary fig. S1, Supplementary Material online), which supported the results of the PCA such that the populations generally showed lower differentiation from those located nearby than those further away (e.g. within and between eastern and western locations). The greatest differentiation was found between the Libyan population and individual Ethiopian populations. Of the Ethiopian populations, Bonga was the most differentiated from the others. A neighbor-joining phylogenetic tree based on Identity-by-State (IBS) estimates between all individuals (supplementary fig. S2, Supplementary Material online) further supports the relationships between populations.

### Environmental Covariates

**Fig. 3:**
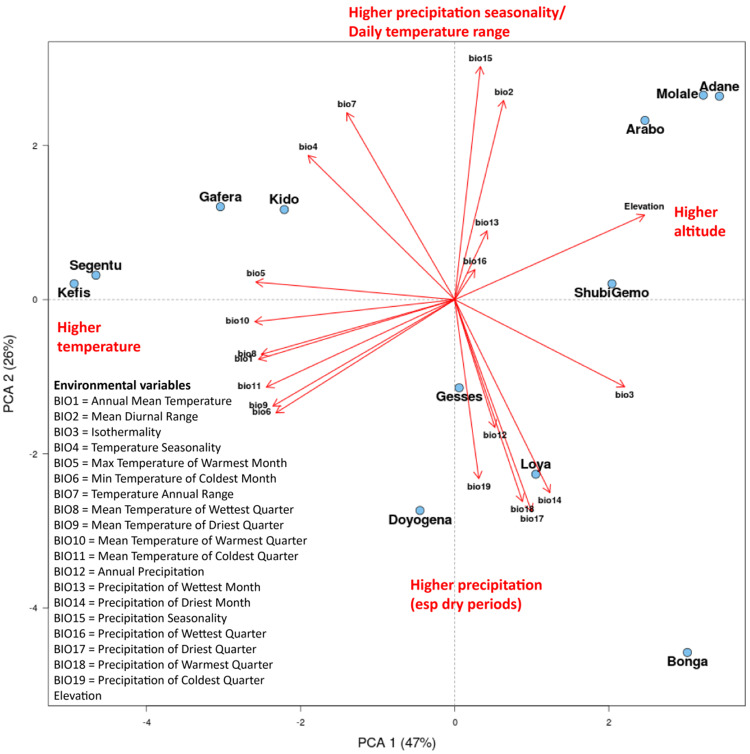
PC1 versus PC2 for PCA of 20 environmental measures of 12 Ethiopian sampling locations.

To clarify the relationships between environmental variables across the Ethiopian study sites, we performed another PCA (fig. 3; scree plot shown in supplementary fig. S3, Supplementary Material online). The first component explained 47% of the variation, which was associated primarily with temperature and altitude such that lower temperature and higher altitude were associated with positive PC1 values. The second principal component accounted for 26% of the variation and was associated with precipitation and seasonality such that higher rainfall and lower seasonality were associated with negative PC2 values. PC3 accounted for 18% of the variation and was also associated with precipitation, such that higher values of rainfall in cold/wet periods were associated with positive values of PC3. One notable point is that the Gesses and Kido sampling locations were separated in the PCA despite geographic proximity because they differed substantially in Elevation and Precipitation of Coldest Quarter measures, due to highly variable local conditions in this region of Ethiopia.

### Signatures of Environmental Adaptation

**Fig. 4:**
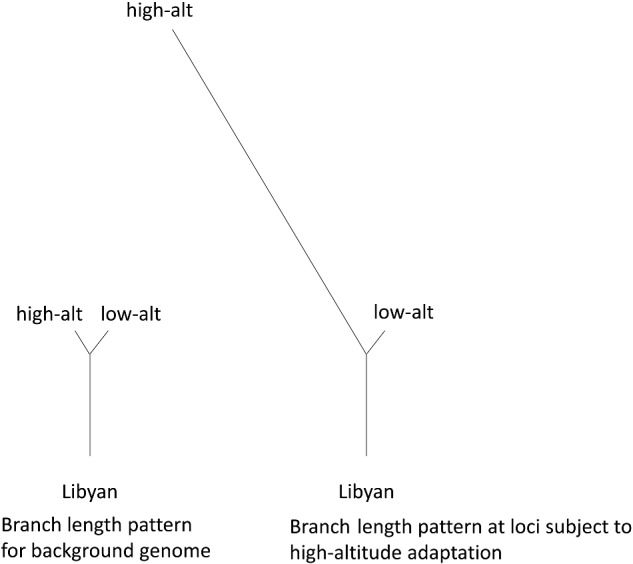
Schematic showing rationale for Population Branch Statistic (PBS) analysis (based on Yi et al. 2010).

**Fig. 5:**
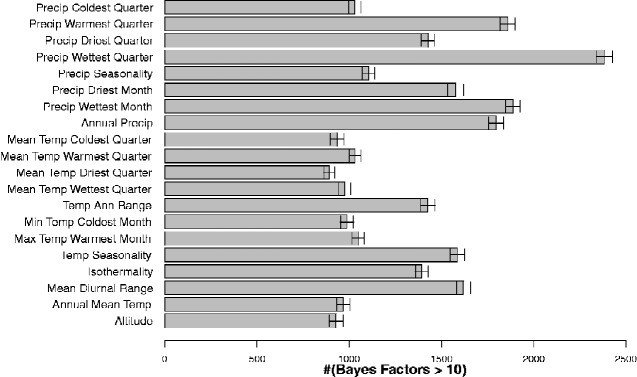
Histogram showing numbers of markers with Bayes factors > 10 (averaged over 100 runs) for Baypass analyses of 20 environmental measures.

Our investigation of the genomic basis of environmental adaptation involved two approaches: (1) specifically regarding high-altitude adaptation, we assessed genetic differentiation between related populations sampled at high and low altitude and (2) considering various environmental factors, we employed an analysis that tests for associations between allele frequencies and environmental variables, as estimated by climatic information of GPS locations. This procedure allowed us to specifically test for environmental adaptation, in contrast to other more general approaches to selection mapping that detect signatures that could be due to various effects, e.g. human-imposed selection, as is likely to be important for livestock. Regarding approach (1), we performed a population-branch statistic (PBS) analysis (Yi et al. 2010), which identifies population-specific allele frequency changes in the high-altitude group of populations (fig. 4) and has been previously applied to examine high-altitude adaptation in humans (Yi et al. 2010) as well as other traits (Fumagalli et al. 2015). As described by Yi et al. (2010), this test relies on comparison of closely-related populations, thus we restricted our analysis to five populations (Adane, Arabo, Menz, Kefis, Segentu), which form the eastern cluster (with high PC1 and PC2 values in fig. 2*a*) and include both high- and low-altitude locations (F_ST_ between pooled low- and high-altitude populations = 0.027). Regarding approach (2) we applied a Bayesian Environmental Association Analysis (Baypass) (Gautier 2015), to detect environment-driven allele frequency differences between populations. Baypass results were run 100 times for each of the 20 environmental measures and Bayes Factors (BFs) were averaged across the 100 runs. The strength of associations between genotype and environmental variables were compared across these variables based on the number of markers with high Bayes Factors (BF > 10) following stringent LD pruning (fig. 5). The environmental trait with the greatest average number of high average BF values was BIO16–Precipitation of Wettest Quarter. Other traits with particularly high values were also related to precipitation (BIO12–Annual Precipitation, BIO13–Precipitation of Wettest Month, and BIO18–Precipitation of Warmest Quarter).

Four environmental variables were selected for further analysis to represent high and low values of the major components of variation between the locations (fig. 3): altitude (high PC1), BIO2 (Mean Diurnal Range, high PC2), BIO5 (Maximum Temperature of Warmest Month, low PC1), BIO12 (Annual Precipitation, low PC2). We also examined results for BIO16 (Precipitation of Wettest Quarter), which had the greatest average count of high BF values.

**Fig. 6:**
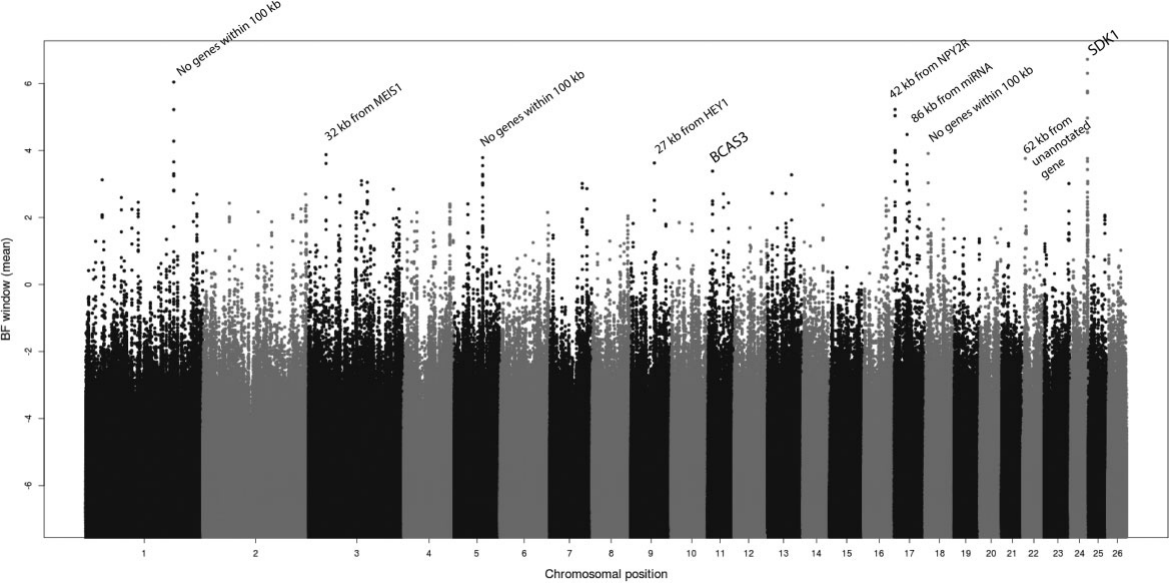
Average (100 Baypass runs) of sliding window mean Bayes Factor values across the genome for Annual Precipitation (BIO12) measure.

To control for random variation at individual sites, means and medians were also calculated for 9-SNP windows across the genome. Thus, three sets of statistics were reported for each of the 6 tests (PBS + five Baypass tests: altitude, BIO2, BIO5, BIO12, BIO16). These 18 statistics included raw values (*PBS raw*, *BF raw* for each of the five Baypass tests), 9-SNP window means (*PBS mean*, *BF mean* x 5) and 9-SNP window medians (*PBS median*, *BF median* x 5). Manhattan plots showing the values of these statistics across the genome are shown in fig. 6 and supplementary figs. S4 (*a*–*c*) – S9 (*a*–*c*), Supplementary Material online. The 56 protein-coding genes directly overlapping the top 0.00001 proportion of SNPs for PBS and Baypass (BF) statistics (table 2) are considered as strong candidates subject to environmental adaptation (genes located within 100 kb of the SNPs are listed in supplementary table S2, Supplementary Material online). Genes directly overlapping the top 0.0001 and top 0.001 proportions of SNPs for PBS and Baypass statistics were also recorded for enrichment analyses, described below (supplementary table S3, Supplementary Material online, gene names including “ORF” were removed).

**Table 2:** List of Protein-Coding Genes Overlapping SNPS Identified in the Top 0.00001 Proportion of PBS and Baypass Results (Raw Values, Mean of 9-SNP Sliding Windows, and Median of 9-SNP Sliding Windows)

Test	Measure	Region	Gene(s)	Raw	Mean	Median
PBS		1: 3613547	*COL6A3*		X	
		6: 65810002	*GABRB1*	X		
		10: 83019595	*TNFSF13B*	X		
		12: 1252208–1254663	*sox13*		X	X
		13: 23128001	*ARMC3*	X		
		13: 60735094–60737256	*TTLL9*	X	X	
		19: 44257952	*ARHGEF3*	X		
		24: 1069638	*CLCN7*, *GNPTG*	X		
Baypass	Altitude	1: 3611207–3614677	*COL6A3*		X	X
		1: 85041405	slc25a24*	X		
		1: 137114512–137114990	*CHODL*	X		
		1: 176389163–176390001	*CFAP44*		X	X
		6: 16572800	*COL25A1*	X		
		6: 45116627–45116714	*SEL1L3*	X		
		10: 49326426–49329922	*KLF12*	X	X	X
		11: 47750060	*ERN1*	X		
		12: 24880669	*SUSD4*			X
		12: 50210421–50251954	*FHAD1*		X	
		12: 62775717	*ARPC5*	X		
		19: 12980756	*MYRIP*	X		
		20: 18875378	*RUNX2*	X		
		23: 3614782–3625866	*ZNF407*		X	X
		24: 40438889	*GNA12*	X		
	BIO2	2: 164700044	*GTDC1*		X	X
		3: 7650048	*wdr34*	X		
		5: 85161518	*TMEM161B*	X		
		11: 14022260–14023453	??	X	X	
		12: 50249074	*FHAD1*	X		
		16: 1644696	*DOCK2*			X
		18: 54417311	*WDR76*	X		
		18: 54472266	*FRMD5*	X		
		19: 17298138	*SRGAP3*	X		
		22: 14992803	*PLCE1*	X		
	BIO5	3: 155499198–155500086	*SRGAP1*	X	X	
		5: 17981229	*DOHH*	X		
		6: 45116627–45116714	*SEL1L3*	X		
		9: 59109369	*EXT1*	X		
		10: 29455988	*eef1a1*, *RXFP2*	X		
		10: 49326426–49329922	*KLF12*	X	X	X
		12: 24880669–24882063	*SUSD4*			X
		12: 50233808	*FHAD1*	X		
		14: 36257047	*WWP2*	X		
		17: 4544219	*fhdc1*	X		
		18: 5985784	*MEF2A*	X		
		23: 3624493	*ZNF407*			X
		24: 40438889	*GNA12*	X		
	BIO12	1: 84016733	*NTNG1*	X		
		1: 128633412	*APP*	X		
		2: 146151992	*KCNH7*	X		
		2: 243930374–243935443	*USP48*			X
		8: 66843820	*ADGRG6*	X		
		8: 75177143	*CCDC170*	X		
		8: 84032474	*PRKN*	X		
		11: 11682275	*BCAS3*		X	
		24: 39452609–39488863	*sdk1*	X	X	X
	BIO16	1: 243634916	*SLC9A9*	X		
		3: 212789216	*CACNA1C*	X		
		11: 11862030–11862364	*BCAS3*	X	X	
		11: 46933828	*TANC2*	X		
		13: 20669196	*PLXDC2*	X		
		18: 21134743	*CRTC3*	X		
		19: 4490590	*RBMS3*		X	
		23: 34985947	*GREB1L*	X		

Genes in small letters indicate that they are not named within the *Ovis aries* v3.1 annotation (for most genes, orthologs were identified in goat or cattle reference genomes, otherwise, indicated by *, orthologs were identified in another reference genome; ?? indicates no gene was identified for any ortholog). Regions indicate the range of SNP positions that overlapped with the named gene. The results cover the PBS analysis and Baypass analyses for altitude, BIO2 (Mean Diurnal Range), BIO5 (Max Temp of Warmest Month), BIO12 (Annual Precipitation), and BIO16 (Precipitation of Wettest Quarter).

The 56 genes overlapping the top 0.00001 SNPs included eight that were identified for more than one environmental variable: *BCAS3* (BIO12, BIO16), *COL6A3* (PBS, altitude), *FHAD1* (altitude, BIO2, BIO5), *GNA12* (altitude, BIO5), *KLF12* (altitude, BIO5), *SEL1L3* (altitude, BIO5), *SUSD4* (altitude, BIO5) and *ZNF407* (altitude, BIO5). Five of these genes were identified by Baypass for both altitude and BIO5 (Max Temperature of Warmest Month).

We used the Variant Effect Predictor Tool (VEPTools) to predict the effect of the top 0.00001 SNPs across the 18 tests (supplementary table S4, Supplementary Material online). Only two SNPs were predicted to have “low,” “moderate,” or “high” consequences on protein function. One SNP identified for PBS mean and PBS median values was predicted to have a “high” consequence on protein function; this was a splice acceptor variant in the *SOX13 gene*. In addition, a SNP identified for the BF raw values for Baypass/BIO5 was predicted to have a moderate consequence on protein function; this was a missense variant (SIFT score = 0.61) in *DOHH*. Forty-one SNPs were identified within noncoding lincRNAs, associated with nine protein-coding genes (table 3, supplementary table S4, Supplementary Material online). The number of these SNPs per environmental variable ranged from one (for altitude and Baypass/BIO12) to 25 (for Baypass/BIO16).

**Table 3:** Noncoding Transcript Exon Variants Found in lincRNA Molecules Overlapping the Genes Encompassing SNPs Identified in the Top 0.00001 Proportion of PBS and Baypass Results (t**able 2**) (Ensembl & VEPtools v98)

lincRNA gene name	Transcript ID	Nearest gene	Test(s)
URS0000A86C59 (RNAcentral)	ENSOART00000028204	*ARMC3*	PBS
U6 spliceosomal RNA [RF00026-AAFC03038941.1/7174-7070]	ENSOART00000026496	*BCAS3*	BIO12, BIO16
URS0000AA49A4 (RNAcentral)	ENSOART00000028035	*CACNA1C*	BIO16
URS0000A900E9 (RNAcentral)	ENSOART00000027871	*GNA12*	BIO5
URS0000A900E9 (RNAcentral)	ENSOART00000028771	*PLCE1*	BIO2
URS0000A88C91 (RNAcentral)	ENSOART00000028203	*PLXDC2*	BIO16
U6 spliceosomal RNA [RF00026-AAFC03112458.1/18651-18546]	ENSOART00000025778	*RBMS3*	BIO16
URS0000A97583 (RNAcentral)	ENSOART00000028897	*RUNX2*	Altitude
URS0000AA3734 (RNAcentral)	ENSOART00000027236	*TMEM161B*	BIO2

Definition of noncoding transcript exon variants: http://www.sequenceontology.org/miso/current_svn/term/SO:0001792.

### Relationship to High-Altitude and Hypoxia-Response Candidate Genes

In order to provide physiological interpretation for the above results, we analysed the relationship between the genes identified by the PBS and Baypass/altitude analyses (both designed to detect associations with high-altitude environments) and genes previously associated with high-altitude adaptation or response to hypoxia. Enrichment of 722 high-altitude candidate genes (compiled from published literature) was tested in the genes overlapping the top 0.0001 and top 0.001 SNPs for the PBS and Baypass/altitude analyses (supplementary table S3, Supplementary Material online) (numbers of genes were too small to assess enrichment for the top 0.00001 SNPs). Genes were pooled for raw value, mean window value and median window value for the respective analyses (table 4). All four sets of results (top 0.001 and top 0.0001 for each PBS and Baypass/altitude) showed enrichment for high-altitude candidate genes, with the top 0.001 sets both significant (p < 0.05) (while tests of the top 0.0001 SNPs were not significant). The top 0.001 of Baypass/altitude results showed the most significant enrichment (p = 2.031e-06, enrichment 2.02-fold). Regarding the overlapping genes, only one high-altitude candidate gene, *ARMC3*, overlapped with top 0.00001 SNPs for either PBS or Baypass/altitude tests (top 0.00001 SNPs for PBS and also top 0.001 SNPs for Baypass/altitude). Another candidate gene, *PRDM16*, overlapped with top 0.0001 SNPS detected by both PBS and Baypass/altitude tests. None of the 163 hypoxia-response candidate genes overlapped with top 0.0001 (or thus top 0.00001) SNPs identified by PBS or Baypass/altitude. There were five hypoxia response candidate genes that overlapped with top 0.001 SNPs identified by PBS (3 genes) or Baypass/altitude (3), including one (*ITPR2*) that was identified by both tests. In addition to *ITPR2*, these genes include *FAM162A*, *GATA6*, *GNGT1*, and *HIF3A*.

**Table 4:** Results of Tests of Enrichment for High-Altitude Candidate Genes (722 Total)

Test	Overlaps	Total	Enrichment	*P* value
PBS				
Top 0.0001	5	68	1.38	0.299
Top 0.001	41	491	1.56	3.04e–3
Baypass: altitude				
Top 0.0001	6	67	1.68	0.147
Top 0.001	49	454	2.02	2.031e–6

### Enrichment of Gene Ontology Terms and Differential Expression across Tissues

In addition to assessing enrichment of high-altitude- and hypoxia-related genes for the PBS and Baypass results, we performed further enrichment investigations. Gene ontology (GO) enrichment was tested in the complete (pooled) set of genes overlapping the top 0.00001 SNPs for the PBS and Baypass (all measures) results (i.e. those listed in table 2; supplementary table S3, Supplementary Material online). These were also evaluated in the genes overlapping the top 0.0001 and top 0.001 (supplementary table S3, Supplementary Material online) SNPs for the individual PBS and Baypass results (numbers of genes were too small to assess enrichment for the top 0.00001 SNPs for individual tests). Differential gene expression across 54 and 30 tissues was assessed for the identified gene sets, based on GTEx RNA-seq data from studies of humans.

For the pooled set of genes overlapping the top 0.00001 SNPs for any of the PBS or Baypass tests, eight biological processes terms and three cellular component terms showed significant enrichment (supplementary table S5, Supplementary Material online). The biological processes terms include “cell morphogenesis involved in neuron differentiation,” “neuron development,” “netrin-activated signaling pathway,” and “biological adhesion.” No molecular function terms showed enrichment. Significant enrichment of differential expression across tissues was also observed. In the comparison of 54 tissues, three artery tissues and five nervous system/brain tissues showed enrichment for up-regulated DEGs and esophagus mucosa showed enrichment for down-regulated DEGs. In the comparison of 30 tissues, nerve, blood vessel and brain showed significant enrichment for up-regulated differentially-expressed genes (DEGs).

Regarding the genes overlapping the top 0.0001 SNPs for the 18 individual PBS and Baypass tests, there were no terms showing significant enrichment of biological processes or molecular functions for any of the gene sets; there was significant enrichment of the cellular component “collagen trimer,” which was enriched in the genes overlapping SNPs associated with BIO2 (Mean Diurnal Range) (BF mean). Regarding the genes overlapping the top 0.001 SNPs for the 18 individual PBS and Baypass tests, there were a number of terms showing significant enrichment for biological processes across the genes associated with different environmental measures (supplementary table S6, Supplementary Material online). The greatest numbers were for BIO5 (Maximum Temperature of Warmest Month) (including terms related to GTPase activity, cell morphogenesis, plasma membrane adhesion, adherens junction organization and neuron projection) and BIO12 (Annual Precipitation) (including terms related to regulation of body fluid levels, coagulation, platelet activation, dendrite development, locomotion, cell adhesion, neuron differentiation, glutamate receptor signalling). Terms related to glutamate receptor signalling also featured in genes associated with BIO2 (Annual Mean Temperature) and BIO16 (Precipitation of Wettest Quarter).

Enrichment of differential expression across tissues was seen for several sets of genes overlapping the top 0.0001 SNPs for the 18 individual PBS and Baypass tests. In the comparison of 54 tissues (supplementary table S7a, Supplementary Material online), artery tissues showed significant enrichment for differentially expressed genes (DEGs) for several tests. There was also evidence of enrichment for DEGs in pituitary, brain cortex, lung and adipose visceral omentum. In the comparison of 30 tissues (supplementary table S7b, Supplementary Material online), there was again evidence for multiple measures of enrichment of DEGs for blood vessel; in addition, there was evidence of enrichment for brain, lung and nerve tissues.

**Fig. 7:**
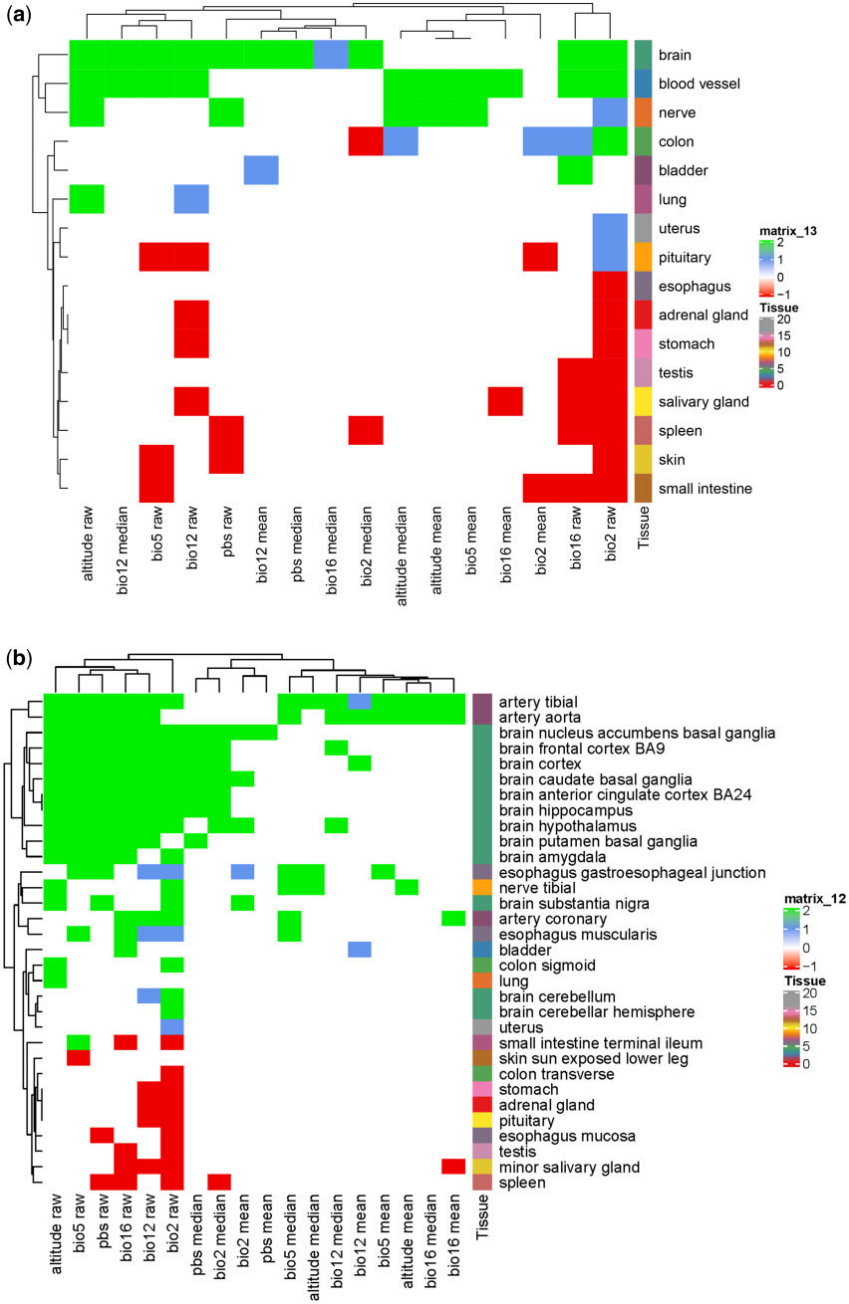
Pattern of enrichment of the top 0.001 gene sets identified by PBS and Baypass among genes up or down regulated in (human-based) GTEx tissues. Gene sets can be enriched specifically among genes up or down regulated in a certain tissue or simply showing differential expression in the tissue relative to others, irrespective of direction. Results are colored according to among which of these categories of differentially expressed genes for each tissue the PBS and Baypass gene sets were enriched. Gene sets enriched among genes downregulated (possibly also showing enrichment among the set of undirected differential expression) in a particular tissue are colored red, those enriched among genes upregulated (again, possibly also showing enrichment among the set of undirected differential expression) in the tissue are colored green and those enriched among genes differentially expressed in the tissue only when ignoring direction are colored blue. Blank (white) cells indicate no enrichment. Tissues that did not show any enrichment are not included in the figure. Hierarchical clustering of gene sets and tissues was based on the extent of sharing of significant hits. (*a*) Comparison of 30 tissues. (*b*) Comparison of 54 tissues.

For most sets of genes overlapping the top 0.001 SNPs, for the 18 individual PBS and Baypass tests, enrichment of differential expression across tissues was also observed. In the comparison of 54 tissues, the tissues that featured in the most tests were related to artery, brain and esophagus (fig. 7*a*, supplementary table S8a, Supplementary Material online). In the comparison of 30 tissues, the tissues that featured in the most tests were brain (11/18 tests) and blood vessel (10/18) (fig. 7*b*, supplementary table S8b, Supplementary Material online). For both sets of tissues (54 and 30), the environmental measure BIO2 (Annual Mean Temperature) included the greatest number of tissues showing differential expression (27/54 and 14/30, respectively).

## Discussion

### Sources of Environmental Selection

Our analysis of genomic variation in Ethiopian sheep showed a stronger association with precipitation-related traits than for temperature- or altitude-related traits in the geographic distribution of genotypes, suggesting a possible role for precipitation-driven selection. This finding is consistent with a large-scale meta-analysis of many species that concluded that precipitation explained a greater proportion of variance in selection than did other climatic factors (Siepielski et al. 2017). If rainfall has imposed selection on Ethiopian sheep, this raises the question of how such selection could be operating. One possible mechanism relates to disease, such as gastrointestinal nematodes, which are a major cause of disease in Ethiopian sheep (Thomas et al. 2007) and their frequency is known to vary with precipitation levels/season (Sissay et al. 2007). Another possible precipitation-related selective force is for drought tolerance, which could cover a further range of phenotypes, e.g. resilience to weight loss during dry seasons, ability to digest/thrive on drought tolerant plants, maintenance of reproductive capacity, disease resilience and behavioural characteristics such as shade usage (Gaughan et al. 2019; Gizaw et al. 2010; Omondi et al. 2008).

There was less evidence for altitude-driven selection in our analysis. This is consistent with less evidence of high-altitude adaptation in human populations from Ethiopia compared to those from Andean or Tibetan regions (Witt and Huerta-Sanchez 2019). It has been suggested that the pattern for humans is due to the less extreme altitudes of the Ethiopian highlands compared to the Andes and Tibet and also to different demographic histories. As sheep are associated with human populations, they might be subject to the same selective pressures related to altitude. Experiments are required to determine if sheep found at high altitude in Ethiopia are physiologically adapted to these conditions. For example, a study of Ethiopian cattle (Wuletaw et al. 2011) found that animals sampled at high altitude had similarly low/moderate pulmonary arterial pressure (PAP) values as those at lower altitude. High PAP values are strong predictors of high-altitude pulmonary hypertension (HAPH), a life-threatening condition; thus low values suggest physiological, and possibly evolutionary, adaptation to high altitude in these animals. The results for Ethiopian cattle are in contrast to the situation for cattle raised in the North American Rocky Mountains, which generally show higher PAP levels at high altitude and are highly prone to HAPH, although there is also evidence of genetic variation in these cattle for propensity to HAPH (Newman et al. 2015).

### Genes/Functions Underlying Environmental Adaptation

To investigate the genomic signatures of environmental adaptation, we looked for overlaps with candidate genes identified by general selection scans in ruminants or by studies focused on specific phenotypes. Two related genes of interest that overlapped with those identified in previous selection scans are *PLCB1* (primarily expressed in brain, Sheep Gene Expression Atlas; Clark et al. 2017a, 2017b) and *PLCE1* (ubiquitously expressed in sheep, Sheep Gene Expression Atlas; Clark et al. 2017a, 2017b), both involved in inositol phosphate metabolism (associated with drought tolerance in various plant species; Pan et al. 2017). *PLCB1* was associated with BIO12 (Annual Precipitation) (top 0.0001) and *PLCE1* was associated with BIO2 (Mean Diurnal Range) (top 0.00001). *PLCE1* was previously identified as a potential selection target by Lv et al (2014) in a study of sheep from a range of environments while *PLCB1* was identified as a potential selection target in several studies: by Kim et al. (2016) in a study of goat and sheep from an Egyptian desert environment, by Taye et al. (2017) in a study of African cattle, and by Li et al. (2020) in a study of Chinese indicine cattle (suggested to have been selected for heat tolerance). We identified four variants with a modifying effect within a lincRNA overlapping the first two exons of *PLCE1*, on the antisense strand. While we can infer little about the functional consequence of these and the other variants identified within lincRNAs in this study, lincRNAs have previously been shown to regulate genes associated with fat tail development in sheep (Bakhtiarizadeh and Salami 2019) and therefore might warrant further investigation.

We also looked for overlaps between genes identified in our study and those associated with specific phenotypes. While fat deposition in the tail is thought to be an adaptation to drought tolerance (similar to the camel hump), there was only a single gene shared between the list of genes identified at highest stringency (top 0.00001) in this study (total = 56 genes) and those identified in a previous study of a similar set of populations, focused on differentiation between populations with different tail morphologies (e.g. thin-tailed, fat-tailed, fat-rump) (total = 177 genes; Ahbara et al. 2019). This gene was *RXFP2*, which is associated with presence and type of horns. In addition, there was no overlap between our list of genes identified at highest stringency and genes linked to signatures of adaptation to extreme environments in Chinese sheep (total = 1180 genes) (Yang et al. 2016), however, there was significantly greater overlap than expected by chance between genes overlapping the top 0.0001 SNPs (total = 127) in our study and genes associated specifically with adaptation to arid environments (total = 376) in the Yang et al (2016) study. The 11 overlapping genes included the *SPTLC3* gene, associated with serum lipid profiles (Zhang et al. 2017), and the coat/skin colour gene *KITLG*, which has also been implicated in temperature adaptation in humans (Yang et al. 2018). There were no overlaps between the genes identified in the current study for any of the environmental measures (at any stringency) and a list of (31) specific candidate genes for nematode-resistance (Sayre and Harris 2012).

As mentioned above, adaptation to high or low precipitation potentially covers a wide range of traits, thus it is particularly challenging to infer how associated genes may be related to selected phenotypes but we explored this by investigating functional information for genes overlapping the top SNPs identified for precipitation-related traits and for enriched pathways and tissue expression. None of the top genes identified by Baypass (those overlapping the top 0.00001 of SNPs) were identified for both of the precipitation-related traits we examined (BIO12, Annual Precipitation, and BIO16, Precipitation of Wettest Quarter). One of the strongest signals we identified was that within the *SDK1* (sidekick cell adhesion molecule 1) gene on OAR24, which was identified for Annual Precipitation using all three measures (BF raw, BF mean and BF median). The protein encoded by *SDK1* is a member of the immunoglobulin superfamily and is highly expressed in artery-aorta and artery-tibial tissues (GTEx; Lonsdale et al. 2013) (it is not present in the Sheep Gene Expression Atlas). SNPs near/in this gene were previously associated with low oxygen saturation in humans from Ethiopian high-altitude regions (Alkorta-Aranburu et al. 2012) and it was identified as a candidate for high altitude/low temperature adaptation in chickens sampled across Ethiopia (Gheyas et al. 2020). It has been associated with a wide range of phenotypes/conditions in humans, including retinal development, brain activity and certain cancers, and with several other traits in livestock, including feed conversion rate in cattle (Barendse et al. 2007), coat color in goats (Nazari-Ghadikolaei et al. 2018) and meat quality of pork (juiciness, intramuscular fat) (Ji et al. 2018).

Genes overlapping the top 0.0001 markers associated with BIO16 (Precipitation of Wettest Quarter) were enriched for differential expression in blood vessel and nerve tissues. Genes overlapping the top 0.001 SNP markers associated with BIO16 and BIO12 (Annual Precipitation) were enriched for differential expression in a number of tissues, again including blood vessel and also brain tissues. Of the five environmental measures we examined, genes overlapping the top 0.001 markers associated with BIO12 were enriched for the greatest number of biological processes and cellular components, which reflected the differential gene expression results in that many were related to cardiovascular or neurological functions. Taken together, these results suggest that adaptation to precipitation is in part related to these functional classes.

Although we saw greater evidence for adaptation to precipitation than temperature, there were some interesting findings regarding the latter. The gene *FHAD1*, associated at highest stringency with both BIO2 (Mean Diurnal Range) and BIO5 (Maximum Temperature of Warmest Month), also showed evidence of temperature adaptation in a study of a wild North American rodent (Garcia-Elfring et al. 2019). Furthermore, two genes (*GLDC* and *LAMC1*) overlapping the top 0.0001 SNPs for both BIO5 and Baypass/altitude were also identified by Flori et al. (2019) as being associated with temperature-related traits in a study of environmental adaptation in Mediterranean cattle. It is not yet clear how the function of these genes relates to thermal adaptation but their detection in multiple studies suggests them as candidates for further study.

Although there was weaker evidence for signatures of adaptation to high-altitude compared to other environmental measures, the genes associated with altitude (detected by PBS or Baypass/altitude) were enriched for the presence of high-altitude candidate genes based on a list of genes identified in previous studies, supporting a role for convergent evolution across distantly-related taxa (Meadows and Lindblad-Toh 2017). The only high-altitude candidate gene overlapping the top 0.00001 PBS or Baypass/altitude SNPs was *ARMC3*, which is involved in regulation of ciliogenesis and thus is potentially related to a range of cilia-related phenotypes across tissues. Three noncoding variants with modifying effects were identified in a lincRNA overlapping *ARMC3* on the antisense strand. This lincRNA is likely to have some regulatory potential due to its proximity to the 5’ proximal region of *ARMC3* (Giral et al. 2018), but this would require further validation. Another high-altitude candidate gene, *PRDM16*, overlapped the top 0.0001 SNPs detected by both PBS and Baypass/altitude. This gene is a regulator of brown and beige fat and thus plays an important role in adipose biology, with implications for energy metabolism (Chi and Cohen 2016). However, none of the genes overlapping the top SNPs identified in this study (top 0.00001 or top 0.0001 for either PBS or Baypass/altitude) are included in the “response to hypoxia” GO annotation list (GO:0001666). This list includes constituents of the hypoxia-inducible factor (HIF) pathway, which has previously been associated with high-altitude adaptation in humans, cattle, yak and other species from Tibet and the Andes (Friedrich and Wiener 2020). Several genes identified at the highest stringency (top 0.00001) were associated with both Baypass/altitude and BIO5 (Max Temperature of Warmest Month), suggesting that some of the adaptation to high altitude may relate to temperature adaptation.

Regarding the genes associated with altitude-related adaptation at the highest stringency (i.e. those overlapping the top 0.00001 of SNPs), *COL6A3* was the only one identified by both PBS and Baypass/altitude. *COL6A3* is highly expressed in many tissues in sheep, with the highest expression in atrioventricular valves (left, right, aortic) and embryonic fibroblasts (Sheep Gene Expression Atlas; Clark et al. 2017a, 2017b). Its highest expression in human is in artery tissues (artery-tibial and artery-aorta, GTEx; Lonsdale et al. 2013). While this gene has not been previously associated with high-altitude adaptation, a number of collagen genes have been, including *COL6A1*, which was identified in at least three separate studies (Alkorta-Aranburu et al. 2012; Azad et al. 2017; Gnecchi-Ruscone et al. 2018). Another collagen gene (*COL25A1*) was also identified by Baypass/altitude. Collagen genes featured notably in a comparison of gene expression in tissues from yak at different altitudes; 5 out of the 14 genes differentially expressed in multiple (>=5) tissues were collagen genes (Qi et al. 2019). Qi et al (2019) argued that this connection could be driven by collagen’s known influence on the behaviour of vascular smooth muscle cells, which are sensitive to hypoxia. None of the genes identified at the highest stringency in the current study (top 0.00001 or top 0.0001) were shared with those reported by Edea et al (2019), who also catalogued signatures of differentiation between Ethiopian sheep from high- and low-altitude regions (the only population shared with this study was Menz). Furthermore, there were no signatures of selection associated with *EPAS1* or *EGLN1*, hypoxia-related genes that have featured in multiple studies of high-altitude adaptation in humans and other species, primarily from Tibet. These genes have also not shown previous evidence of selection in Ethiopian populations of humans or other species and, as mentioned above, it has been suggested that the selection pressure may be lower in Ethiopia highlands compared to Tibet and the Andes due to the slightly lower altitude (Witt and Huerta-Sanchez 2019).

In summary, we used a landscape genomic approach to examine associations between markers and environmental variables for sampling locations of native Ethiopian sheep. Using this approach, we determined which climatic variables showed the strongest association with allele frequency variation in the sheep genome. This allowed us to identify candidate loci associated with particular environmental characteristics. These results suggest that adaptation to precipitation levels has had a greater impact on the genome than altitude or temperature. There was however also evidence of enrichment for genes previously associated with high-altitude adaptation although no strong association for altitude was identified with hypoxia inducible factor (HIF) genes (e.g. *EPAS1* and *EGLN1*). We highlight examples of candidate loci potentially associated with environmental measures and their tissue-specific expression patterns in sheep, e.g. *PLCB1*. These loci are suitable candidates for experimental functional validation in further studies. This study provides a foundation to investigate further the effects of climatic variables on small ruminant populations.

## Materials and Methods

### Sheep Populations

A set of 13 sheep populations were analysed in this study, 12 from a range of environmental conditions and geographical regions across Ethiopia and one from Libya (LBR), used as an outgroup for some analyses (table 1, fig. 1). These include the same Ethiopian populations as described in Ahbara et al. (2019) (their table 1), with one additional population, Segentu.

### Library Preparation and Whole-Genome Sequencing

High quality genomic data were extracted from ear notch biopsies for 130 sheep using a Nucleospin Tissue Kit and quality checked using an Agilent Tapestation 2200. For library preparation, 1 µg of gDNA was sheared to fragments of 450 bp mean size using a Covaris LE220 focused-ultrasonicator. DNA fragments were blunt ended, A-tailed, size selected and adapters ligated onto fragment ends according to Illumina TruSeq PCR-free library preparation kit protocol. Insert size on the libraries was evaluated using a PerkinElmer LapChip GX Touch with an HT DNA 1k/12K/HI SENS LabChip and HT DNA HI SENS Reagent Kit. Final library concentration was calculated by qPCR using a Roche LightCycler 480 and a Kapa Illumina Library Quantification kit and Standards. Then libraries were normalized to a loading concentration of 150 nM. All the library processing steps were carried out on Hamilton MicroLab STAR liquid handling robots coupled to BaseSpace Clarity LIMS X Edition. Libraries for all samples were loaded into a HiSeq X Flow cell v2.5, and clustered using an Illumina cBot2 Cluster Generation System. All libraries were sequenced on the HiSeqX to a mean coverage of 54X with 150 bp paired-end reads.

### Demultiplexing and Trimming

Demultiplexing was performed using bcl2fastq (v.2.17.1.14), allowing 1 mismatch when assigning reads to barcodes. Adapters (Read1:AGATCGGAAGAGCACACGTCTGAACTCCAGTCA, Read2:AGATCGGAAGAGCGTCGTGTAGGGAAAGAGTGT) were trimmed during the demultiplexing process. After trimming and demultiplexing, two compressed FASTQ files (“fastq.gz”) for each sample were obtained.

### Sequence Mapping

The *Ovis aries* (sheep) genome (v3.1) produced by the International Sheep Genome Consortium (ISGC) was downloaded from Ensembl release 88 (ftp://ftp.ensembl.org/pub/release-88/fasta/ovis_aries/dna/Ovis_aries.Oar_v3.1.dna.toplevel.fa.gz). Clean reads for all 130 WGS samples were mapped to the *O. aries* genome using the Burrows-Wheeler Alignment tool (BWA-MEM) version bwa-0.7.12-r1039 (Li 2013; Li and Durbin 2009). The alignment files generated in SAM format were converted to BAM format using SAMtools v.1.19 (Li et al. 2009).

### Variant Calling

We then applied the Best Practices preprocessing Genome Analysis Toolkit (GATK) v3.7 workflow from the Broad Institute to perform variant discovery (https://software.broadinstitute.org/gatk/best-practices). The alignment files were sorted by coordinate and indexed using SAMTools. The Picard suite of tools v.1.139 (http://sourceforge.net/projects/picard) was used to mark duplicate reads. Base Quality Score Recalibration (BQSR) was performed using BaseRecalibrator from the Genome Analysis Toolkit (GATK v.3.7) (McKenna et al. 2010) with the “knownSites” set to the *O. aries* dbSNP from Ensembl release 88 (ftp://ftp.ensembl.org/pub/release-88/variation/vcf/ovis_aries/Ovis_aries.vcf.gz).

Variants were called using HaplotypeCaller from GATK (with -ERC GVCF and -stand_call_conf set to 30) followed by GenotypeGVCFs to perform joint genotyping and generate the VCF files containing SNPs and Indels for all samples. Variant quality score recalibration was performed using VariantRecalibrator with the following parameters: -an QD -an MQ -an MQRankSum -an ReadPosRankSum -an FS -an SOR -an DP -an InbreedingCoeff and the resource parameters set as: resource: eva, known=false, training=true, truth=true, prior=15.0 evaFile and resource: dbsnp, known=true, training=false, truth=false, prior=2.0 dbsnpFile, where “evaFile” and “dbsnpFile” refer to the set of high quality variants from the European Variation Archive (ftp://ftp.ebi.ac.uk/pub/databases/eva/PRJEB14685/eva_normalised_files/*.filtered_intersect.vcf.gz) and the dbSNP VCF file from Ensembl release 88 (ftp://ftp.ensembl.org/pub/release-88/variation/vcf/ovis_aries/Ovis_aries.vcf.gz) for *O. aries*, respectively.

### Quality Control Procedures

Markers were filtered using vcftools for the following criteria: marker quality (–minQ 40), overall missingness (–max-missing 0.8), deviations from HWE (–hwe 0.00000001), allele count (–min-alleles 2, –max-alleles 2) and minor allele frequency (–maf 0.05). Next we used PLINK (Chang et al. 2015; Purcell and Chang) to produce a pruned subset of markers in approximate linkage equilibrium (using –indep-pairwise option, with window size = 500 kb, step size = 1, r^2^ threshold = 0.8). Samples (27) were then removed such that there were no close relatives (first- or second-degree, using vcftools –relatedness2 option) in the data set, leaving 103 individuals across the 13 populations (94 across the 12 Ethiopian populations), ranging from 4 to 10 per population. Finally, markers were removed that had genotypes for fewer than 80% of individuals within a population or were fixed for a single allele across the 12 Ethiopian populations. The final data set comprised 3,237,954 markers. All subsequent analyses were performed on autosomes, comprising 3,095,833 markers. Average per-SNP diversity within each population was calculated using vcftools.

### Predicted Functional Consequences of Variants

We used Ensembl Variant Effect Predictor (VEPtools v98; McLaren et al. 2016) to predict the effects of the variants, including the “–sift b” and “–nearest symbol” options. The noncoding variants most likely to impact phenotype, transcript exon variants (e.g. lincRNAs) for SNPs associated with annotated genes, were extracted from the VEPtools output for further analysis.

### Environmental Variables

Altitude and the standard 19 WorldClim bioclimatic variables (BIO1–BIO19) (Fick and Hijmans 2017; WorldClim) were recorded for each of the locations where the 12 Ethiopian sheep populations were sampled (table 1). A PCA was performed on the 20 variables for the 12 Ethiopian locations using the prcomp command within R (variables scaled and centered to zero).

### PCA of Individuals

PCA was performed on the genomic data for the 12 Ethiopian sheep populations, both with and without the Libyan population (LBR) as an outgroup, using PLINK (Chang et al. 2015; Purcell and Chang) with its default options.

### Phylogenetic Reconstruction

Identity-by-state estimates of genetic distance were calculated for all pairs of (103) individuals using PLINK (Chang et al. 2015; Purcell and Chang). A neighbor-joining tree (Saitou and Nei 1987) was reconstructed based on these distances using Phylip (Felsenstein 2005).

### Population Differentiation

The population-branch statistic (PBS) (Yi et al. 2010) is designed to identify population-specific allele frequency changes, in this case, to identify alleles associated with adaptation to high altitude. A subset of five closely related populations sampled at high- and low-altitude (high, 2,610–2,783 m: AKD, AKR, MZ; low, 740–859 m: FKD, FSG) was selected for analysis in order to limit overall between-population differences, as suggested by Yi et al. (2010). The three high- and two low-altitude populations were pooled into two groups in order to improve the power of the analysis. PBS, a function of pairwise *F*_ST_ values, was calculated on these populations and the population from Libya (LBR) as an outgroup. First, pairwise *F*_ST_ (Weir and Cockerham 1984) was calculated for all markers between the pooled high-altitude, pooled low-altitude and LBR populations using vcftools (option: –weir-fst-pop) (https://vcftools.github.io/index.html) (Danecek et al. 2011). Negative *F*_ST_ values were set to 0. PBS (*PBS raw*) was calculated using these *F*_ST_ values, as described in Yi et al. (2010). To control for random variation at individual sites, means and medians of PBS (*PBS mean*, *PBS median*) were also calculated for 9-SNP windows across the genome. This window definition was found to be most or equally suitable for estimation of local genomic diversity, by balancing capture of extreme signals and removal of stochastic effects, of in comparison with 11- and 13-SNP windows or windows based on physical size (results not shown). The number of markers for which *F*_ST_ was calculated (3,068,678) was reduced from the initial data set due to fixation of the same allele across the five populations (but not across all of the 12 populations, as filtered in QC procedures). The number of markers was further reduced for the PBS analysis since additional markers were removed that were missing *F*_ST_ values for high-altitude versus LBR or low-altitude versus LBR comparisons. Final numbers of markers were the following: 3,007,909 (PBS raw) and 2,421,841 (PBS mean and PBS median). Genes located less than 100 kb of SNPs in the top 0.00001 proportion of PBS statistics were catalogued. Genome-wide average pairwise *F*_ST_ values (Weir and Cockerham 1984) were also calculated for all pairs of populations using vcftools, as described above. Negative *F*_ST_ values were again set to 0. An unrooted neighbor-joining tree (Saitou and Nei 1987) was reconstructed based on these F_ST_ values using Phylip (Felsenstein 2005).

### Genotype-Environment Association

Baypass (Gautier 2015) was used to identify genetic loci associated with population-specific covariates, in this case, the 20 environmental characteristics of the locations where the sheep populations were sampled. This was implemented under the IS covariate mode (*STD covariate model*), as described in the manual (5.1.2), specifying the omega matrix (covariance matrix of population allele frequencies) for the entire marker set, which was calculated from a separate Baypass run. To account for the variation between MCMC runs, Baypass was run 100 times, each with a randomly-chosen seed. In each run, a Bayes Factor in deciban (dB) units [“BF” = 10×log_10_(BF)] was calculated for each genotype x environmental variable combination and these were averaged over the 100 runs to produce average BFs (*BF raw*) for each combination of marker position and environmental measure. As for the PBS analyses, means and medians of BF values were also calculated for 9-SNP windows (*BF mean* and *BF median*, respectively) centered on a total of 3,130,759 markers across the genome. Genes within 100 kb of SNPs in the top 0.00001 proportion of average BF statistics were catalogued.

In order to compare evidence for selection due to different environmental factors, we also compared the number of high BF values across the 20 environmental variables. We first carried out additional pruning to further reduce the linkage disequilibrium (LD) between markers (using PLINK, as described above but with *r*^2^ threshold = 0.5). From this pruned set of markers (1,434,184), the number of BF values >10 for each run and each environmental variable was counted and counts were then averaged across the 100 Baypass runs.

### Relationship to High-Altitude and Hypoxia-Related Candidate Genes

Enrichment of high-altitude candidate genes was tested for the genes identified by PBS and Baypass (high-altitude) analyses. First, a list of 722 autosomal candidate genes (supplementary table S9, Supplementary Material online) was compiled by surveying the literature on high-altitude adaptation in humans and other species and including all genes that were identified in at least two studies. Secondly, a list of 163 genes associated with “response to hypoxia” (GO:0001666) was compiled. Genes were first extracted from the Mouse Genome Informatics resource [Mouse Genome Database (MGD)]. Then gene names were converted to HGNC/Hugo names using HGNC’s Multi-symbol checker (HGNC Database). For both lists, genes were filtered for their presence on autosomes within the sheep genome using Biomart/Ensembl (Hunt et al. 2018). To enable enrichment testing, the total number of autosomal protein-coding genes with HGNC symbols for the *O. aries* genome assembly version 3.1 (Oar_v3.1) was determined using Biomart/Ensembl as 13,510. Gene names including “ORF” were excluded because their function was unknown. Next, the genes overlapping between the lists of candidates and those identified by PBS/Baypass analyses were counted. Enrichment of the candidate genes within the PBS/Baypass results was assessed based on the cumulative distribution function (CDF) of the hypergeometric distribution.

### Testing GO and Tissue Expression Enrichment of Gene Sets

Sets of genes identified by Baypass/PBS were assessed for enrichment of GO terms (biological processes, cellular components, molecular functions) compared to the full list of *O. aries* autosomal protein-coding genes described above using the GENE2FUNC option in FUMA (Watanabe et al. 2017), using default parameters. Significance was assessed based on FDR-adjusted p-value. These gene sets were also tested for overrepresentation in sets of differentially expressed genes from (human-based) GTEx v8 RNA-seq data of 54 and 30 tissues (Lonsdale et al. 2013). Relationships between the differential expression patterns of SNP sets and tissues were determined using hierarchical clustering by extent of sharing of significant hits and visualized using complex heatmaps (Gu et al. 2016).

## Supplementary Material

Supplementary data are available at *Genome Biology and Evolution* online.

## Supplementary Material

evab014_Supplementary_Data
