# Supplementary material for: Whole-Genome Sequence Data Suggest Environmental Adaptation of Ethiopian Sheep Populations
Source: Genome Biol Evol. 2021 Jan 27;13(3):evab014. doi: 10.1093/gbe/evab014 (PMC7955157; doi:10.1093/gbe/evab014)
Supplement: evab014_Supplementary_Data [file evab014_supplementary_data.zip › Supplementary Legends.docx]

**Supplementary Material**

Table S1. Average pairwise F_ST_ across 13 populations (12 Ethiopian populations and 1 Libyan outgroup, LBR).

Table S2. Genes located within 100 kb of top proportion of 0.00001 SNPs identified by PBS and Baypass analyses (individual tests).

Table S3. Gene sets used for enrichment analyses: pooled set of genes directly overlapping the top proportion of 0.00001 SNPs identified by all PBS and Baypass analyses and sets of genes directly overlapping top 0.0001 and top 0.001 SNPs identified by individual PBS and Baypass analyses.

Table S4. Output from VEPtools for annotated gene-associated variants in the top 0.00001 proportion of SNPs identified by PBS and Baypass analyses.

Table S5. Significant enrichment terms for genes overlapping top 0.00001 PBS or Baypass (all measures) results (pooled across all tests).

Table S6. Enrichment of biological processes for top 0.001 gene sets (individual tests).

Table S7. Enrichment of differential expression across tissues (based on GTEx) for top 0.0001 gene sets (individual tests). a. 54 tissues. b. 30 tissues.

Table S8. Enrichment of differential expression across tissues (based on GTEx) for top 0.001 gene sets (individual tests). a. 54 tissues. b. 30 tissues.

Table S9. List of autosomal high-altitude adaptation candidate genes from published literature.

Figure S1: Neighbour-joining phylogenetic tree based on average F_ST_ values between 13 sheep sheep populations (Ethiopian: AKD, AKR, BO, DA, FKD, FSG, GGD, KO, LA, MZ, SHG, WA; Libyan: LBR).

Figure S2: Neighbour-joining phylogenetic tree based on Identity-by-State (IBS) estimates between all (103) individuals. Orange indicates high-altitude populations (AKD, AKR, MZ), green indicates low-altitude populations (FKD, FSG) and red indicates the Libyan population (LBR).

Figure S3: Scree plot for PCA of environmental measures across 12 Ethiopian sampling sites (see Table 1 for description).

Figures S4-S9: Manhattan plots for all PBS and Baypass analyses. S4: PBS; S5: Baypass/altitude; S6: Baypass/BIO2; S7: Baypass/BIO5; S8: Baypass/BIO12; S9: Baypass/BIO16 (a. raw, b. mean, c. median)
