# Supplementary material for: Whole-Genome Sequence Data Suggest Environmental Adaptation of Ethiopian Sheep Populations
Source: Genome Biol Evol. 2021 Jan 27;13(3):evab014. doi: 10.1093/gbe/evab014 (PMC7955157; doi:10.1093/gbe/evab014)

Supplementary Table S1. Average pairwise F_{ST} across 13 populations (12 Ethiopian populations and 1 Libyan outgroup, LBR).

	AKR	BO	DA	FKD	FSG	GGD	KO	LA	LBR	MZ	SHG	WA
AKD	0.035	0.076	0.055	0.040	0.052	0.058	0.060	0.067	0.058	0.037	0.052	0.052
AKR		0.079	0.059	0.030	0.043	0.062	0.063	0.070	0.056	0.050	0.055	0.059
BO			0.057	0.082	0.101	0.056	0.059	0.071	0.095	0.081	0.066	0.068
DA				0.062	0.077	0.049	0.051	0.047	0.077	0.060	0.036	0.048
FKD					0.041	0.065	0.067	0.073	0.060	0.058	0.058	0.063
FSG						0.079	0.079	0.090	0.069	0.071	0.072	0.078
GGD							0.040	0.065	0.076	0.065	0.054	0.051
KO								0.068	0.077	0.067	0.056	0.053
LA									0.087	0.074	0.038	0.064
LBR										0.066	0.073	0.075
MZ											0.058	0.053
SHG												0.052

Supplementary Table S5. Significant enrichment terms for genes overlapping top 0.00001 PBS or Baypass (all measures) results (pooled across all tests).

**Biological processes terms**	**adj p-value**		**Cellular components terms**	**adj p-value**
Cell morphogenesis involved in differentiation	2.34E-04		Synapse	4.20E-02
Netrin activated signalling pathway	3.74E-03		Cell cortex	4.20E-02
Cell morphogenesis involved in neuron differentiation	3.74E-03		Cytoplasmic region	4.48E-02
Cellular component morphogenesis	8.34E-03			
Cell part morphogenesis	1.57E-02			
Neuron development	2.16E-02			
Regulation of NMDA receptor activity	3.29E-02			
Biological adhesion	4.92E-02			

Supplementary Table S6. Enrichment of biological processes for top 0.001 gene sets (individual tests).

		**biological processes**	**cellular components**	**molecular functions**
**PBS**	**raw**	biological_adhesion	neuron_part	
		cell_morphogenesis_involved_in_differentiation	neuron_projection	
			basement_membrane	
			synapse	
			cell_junction	
			cell_cell_adherens_junction	
			glutamatergic_synapse	
**altitude**	**raw**		synapse	
	**median**			transmembrane_receptor_protein_phosphatase_activity
**BIO2**	**raw**		synapse	
			synapse_part	
			presynapse	
	**mean**	ionotropic_glutamate_receptor_signaling_pathway		ionotropic_glutamate_receptor activity
				glutamate_receptor activity
				postsynaptic_neurotransmitter_receptor_activity
				transmitter_gated_channel_activity
**BIO5**	**raw**	regulation_of_GTPase_activity	laminin_complex	
		adherens_junction_organization		
		cell_cell_adhesion_via_plasma_membrane_adhesion_molecules		
		cell_morphogenesis_involved_in_differentiation		
		neuron_projection_guidance		
		positive_regulation_of_GTPase_activity		
		cellular_component_morphogenesis		
	**mean**		laminin_complex	
**BIO12**	**raw**	biological_adhesion	neuron_to_neuron_synapse	
		regulation_of_body_fluid_levels	postsynapse	
		coagulation	synapse	
		taxis	postsynaptic_membrane	

		platelet_activation	postsynaptic_density_membrane	
		regulation_of_GTPase_activity	neuron_part	
		dendrite_development	postsynaptic_specialization_membrane	
		cell_morphogenesis_involved_in_neuron_differentiation	glutamatergic_synapse	
		locomotion	cell_junction	
		cell_morphogenesis_involved_in_differentiation	intrinsic_component_of_postsynaptic_density_membrane	
		glutamate_receptor_signaling_pathway	cell_cell_junction	
		negative_regulation_of_cell_substrate_adhesion	extrinsic_component_of_postsynaptic_density_membrane	
		regulation_of_cell_adhesion	excitatory_synapse	
		cell_cell_adhesion	plasma_membrane_region	
			neuron_projection	
			extrinsic_component_of_postsynaptic_specialization_membrane	
			basement_membrane	
			intrinsic_component_of_postsynaptic_specialization_membrane	
			presynapse	
			presynaptic_membrane	
			intrinsic_component_of_synaptic_membrane	
			podosome	
			extrinsic_component_of_synaptic_membrane	
**BIO16**	**raw**	glutamate_receptor_signaling_pathway	synapse	ionotropic_glutamate_receptor activity
			synapse_part	glutamate_receptor activity
			neuron_to_neuron_synapse	
			neurotransmitter_receptor_complex	
			neuron_part	
			glutamatergic_synapse	

Supplementary Table S7a. Enrichment of differential expression across tissues (based on GTEx) for top 0.0001 gene sets (individual tests) (54 tissues).

	**raw**	**mean**	**median**
**altitude**		artery_tibial	artery_tibial
**PBS**	adipose_visceral_omentum		
**BIO2**	brain_cortex	pituitary	
**BIO5**	artery_tibial<br>artery_aorta	lung<br>artery_tibial	artery_tibial
**BIO12**			
**BIO16**	artery_tibial<br>artery_aorta		

Supplementary Table S7b. Enrichment of differential expression across tissues for top 0.0001 gene sets (GTEx: 30 tissues).

	**raw**	**mean**	**median**
**altitude**			blood_vessel
**PBS**			
**BIO2**	brain		
**BIO5**		lung<br>blood_vessel	
**BIO12**			
**BIO16**	nerve<br>blood_vessel		

Supplementary Table S8a. Enrichment of differential expression across tissues (based on GTEx) for top 0.001 gene sets (individual tests) (54 tissues).

	raw	mean	median
**altitude**	artery_aorta<br>artery_tibial<br>nerve_tibial<br>brain_frontal_cortex_BA9<br>brain_hippocampus<br>brain_nucleus_accumbens_basal_ganglia<br>lung<br>brain_cortex<br>brain_caudate_basal_ganglia<br>brain_hypothalamus<br>brain_amygdala<br>colon_sigmoid<br>brain_anterior_cingulate_cortex_BA24<br>brain_substantia_nigra<br>brain_putamen_basal_ganglia	artery_aorta<br>artery_tibial<br>nerve_tibal	nerve_tibial<br>esophagus_gastroesophageal_junction<br>artery_tibial
**PBS**	artery_aorta<br>brain_frontal_cortex_BA9<br>artery_tibial<br>brain_nucleus_accumbens_basal_ganglia<br>esophagus_gastroesophageal_junction<br>brain_hypothalamus<br>brain_cortex<br>spleen<br>brain_anterior_cingulate_cortex_BA24<br>esophagus_mucosa<br>brain_caudate_basal_ganglia<br>brain_substantia_nigra<br>brain_hippocampus<br>brain_amygdala<br>brain_putamen_basal_ganglia	brain_nucleus_accumbens_basal_ganglia	brain_nucleus_accumbens_basal_ganglia<br>brain_frontal_cortex_BA9<br>brain_putamen_basal_ganglia<br>brain_hippocampus<br>brain_cortex<br>brain_caudate_basal_ganglia<br>brain_anterior_cingulate_cortex_BA24
**BIO2**	brain_cerebellum<br>nerve_tibial	brain_nucleus_accumbens_basal_ganglia<br>brain_hypothalamus	brain_nucleus_accumbens_basal_ganglia<br>brain_frontal_cortex_BA9

	pituitary<br>colon_sigmoid<br>artery_tibial<br>brain_frontal_cortex_BA9<br>brain_cerebellar_hemisphere<br>esophagus_gastroesophageal_junction<br>brain_cortex<br>esophagus_muscularis<br>artery_coronary<br>colon_transverse<br>esophagus_mucosa<br>adrenal_gland<br>brain_hypothalamus<br>uterus<br>brain_anterior_cingulate_cortex_BA24<br>minor_salivary_gland<br>small_intestine_terminal_ileum<br>stomach<br>brain_hippocampus<br>brain_nucleus_accumbens_basal_ganglia<br>testis<br>brain_substantia_nigra<br>brain_caudate_basal_ganglia<br>spleen<br>brain_amygdala	brain_caudate_basal_ganglia<br>brain_substantia_nigra<br>esophagus_gastroesophageal_junction	brain_anterior_cingulate_cortex_BA24<br>brain_hypothalamus<br>brain_cortex<br>spleen<br>brain_caudate_basal_ganglia<br>brain_hippocampus
**BIO5**	artery_aorta<br>artery_tibial<br>small_intestine_terminal_ileum<br>brain_frontal_cortex_BA9<br>esophagus_gastroesophageal_junction<br>esophagus_muscularis<br>brain_nucleus_accumbens_basal_ganglia<br>brain_cortex<br>brain_hypothalamus	artery_aorta<br>artery_tibial<br>esophagus_gastroesophageal_junction	esophagus_gastroesophageal_junction<br>artery_aorta<br>nerve_tibial<br>artery_tibial<br>esophagus_muscularis<br>artery_coronary

	skin_sun_exposed_lower_leg<br>brain_caudate_basal_ganglia<br>brain_hippocampus<br>brain_amygdala<br>brain_anterior_cingulate_cortex_BA24<br>brain_putamen_basal_ganglia		
**BIO12**	artery_aorta<br>artery_coronary<br>artery_tibial<br>esophagus_muscularis<br>brain_frontal_cortex_BA9<br>esophagus_gastroesophageal_junction<br>brain_cerebellum<br>brain_cortex<br>adrenal_gland<br>pituitary<br>minor_salivary_gland<br>stomach<br>brain_nucleus_accumbens_basal_ganglia<br>brain_hypothalamus<br>brain_caudate_basal_ganglia<br>brain_anterior_cingulate_cortex_BA24<br>brain_hippocampus<br>brain_putamen_basal_ganglia	artery_tibial<br>bladder<br>artery_aorta<br>brain_cortex	artery_aorta<br>artery_tibial<br>brain_hypothalamus<br>brain_frontal_cortex_BA9
**BIO16**	artery_tibial<br>artery_coronary<br>artery_aorta<br>bladder<br>minor_salivary_gland<br>brain_cortex<br>esophagus_muscularis<br>small_intestine_terminal_ileum<br>brain_frontal_cortex_BA9<br>testis	artery_tibial<br>artery_aorta<br>artery_coronary<br>minor_salivary_gland	artery_tibial<br>artery_aorta

	brain_hypothalamus<br>spleen<br>brain_hippocampus<br>brain_nucleus_accumbens_basal_ganglia<br>brain_caudate_basal_ganglia<br>brain_anterior_cingulate_cortex_BA24<br>brain_putamen_basal_ganglia<br>brain_amygdala		

Supplementary Table S8b. Enrichment of differential expression across tissues (based on GTEx) for top 0.001 gene sets (individual tests) (30 tissues).

	raw	mean	median
**altitude**	nerve<br>brain<br>blood_vessel<br>lung	nerve<br>blood_vessel	nerve<br>blood_vessel<br>colon
**PBS**	nerve<br>brain<br>skin<br>spleen		brain
**BIO2**	nerve<br>pituitary<br>colon<br>brain<br>esophagus<br>adrenal_gland<br>blood_vessel<br>uterus<br>salivary_gland<br>small_intestine<br>stomach<br>skin<br>testis<br>spleen	pituitary<br>small_intestine<br>colon	colon<br>brain<br>spleen
**BIO5**	brain<br>pituitary<br>blood_vessel<br>small_intestine<br>skin	nerve<br>blood_vessel	
**BIO12**	blood_vessel<br>lung<br>brain<br>adrenal_gland<br>pituitary	bladder<br>brain	blood_vessel<br>brain

	salivary_gland<br>stomach		
**BIO16**	bladder<br>colon<br>blood_vessel<br>salivary_gland<br>small_intestine<br>testis<br>brain<br>spleen	blood_vessel<br>salivary_gland	brain

Fig S1. Neighbour-joining phylogenetic tree based on average F_{ST} values between 13 sheep populations (Ethiopian: AKD, AKR, BO, DA, FKD, FSG, GGD, KO, LA, MZ, SHG, WA; Libyan: LBR).

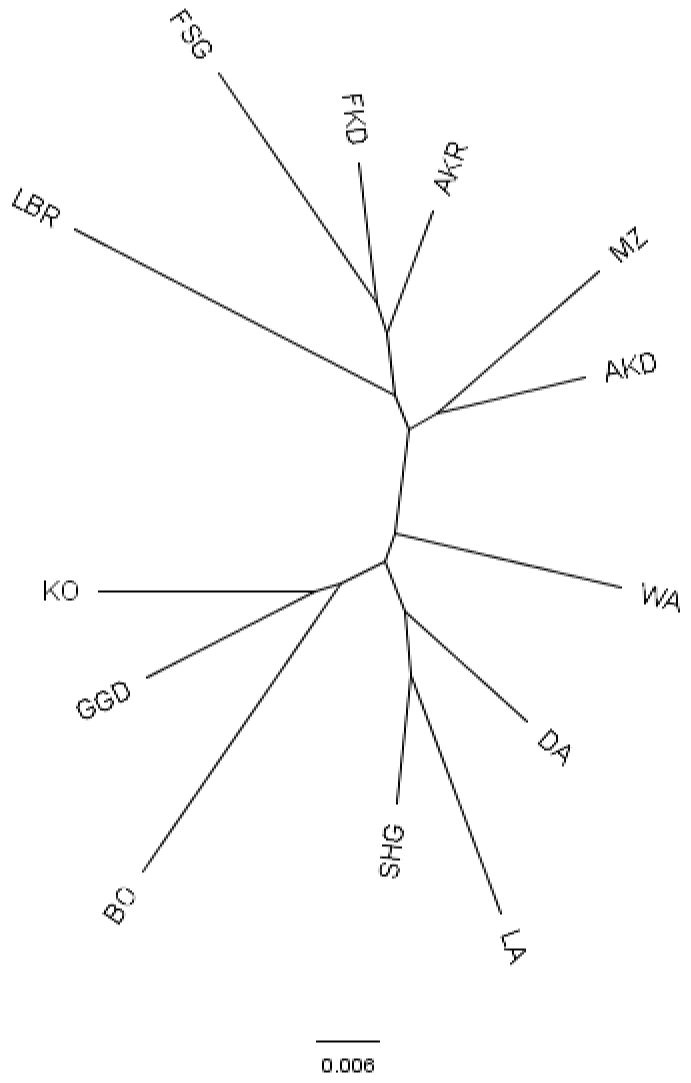

Fig S2. Neighbour-joining phylogenetic tree based on Identity-by-State (IBS) estimates between all (103) individuals. Orange indicates high-altitude populations (AKD, AKR, MZ), green indicates low-altitude populations (FKD, FSG) and red indicates the Libyan population (LBR).

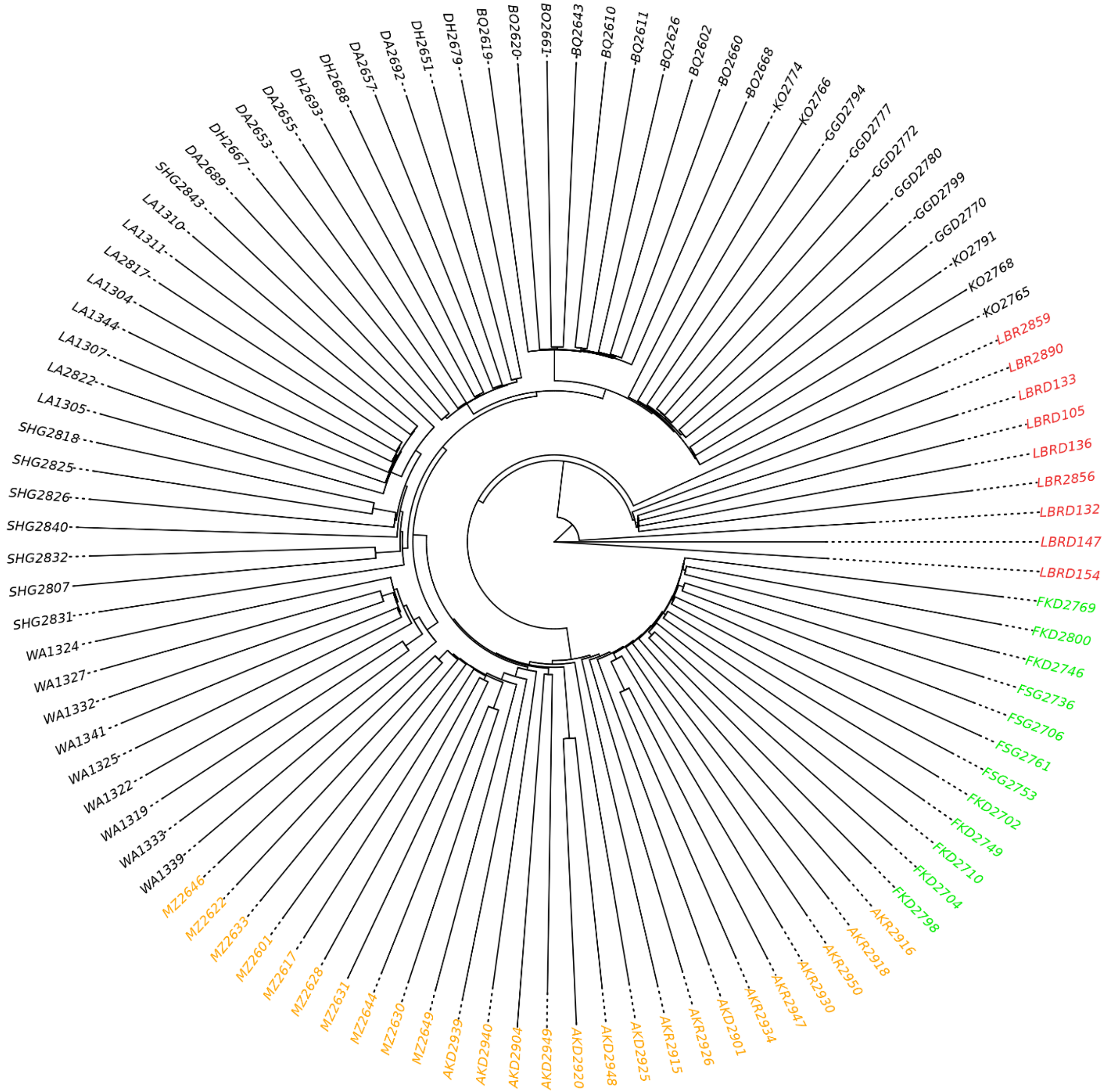

Fig S3. Scree plot for PCA of environmental measures across 12 Ethiopian sites (see Table 1).

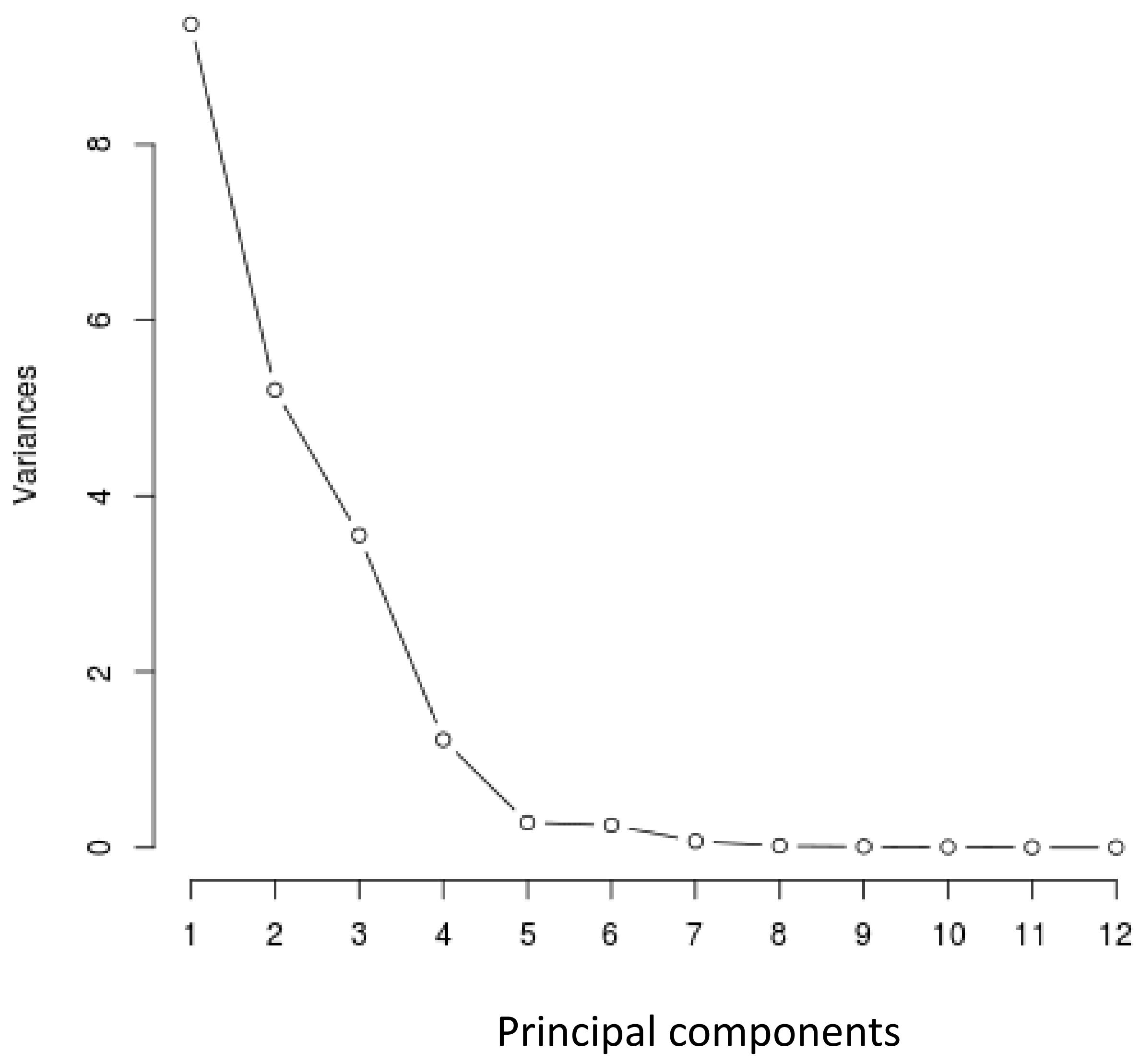

Fig S4a. PBS: raw

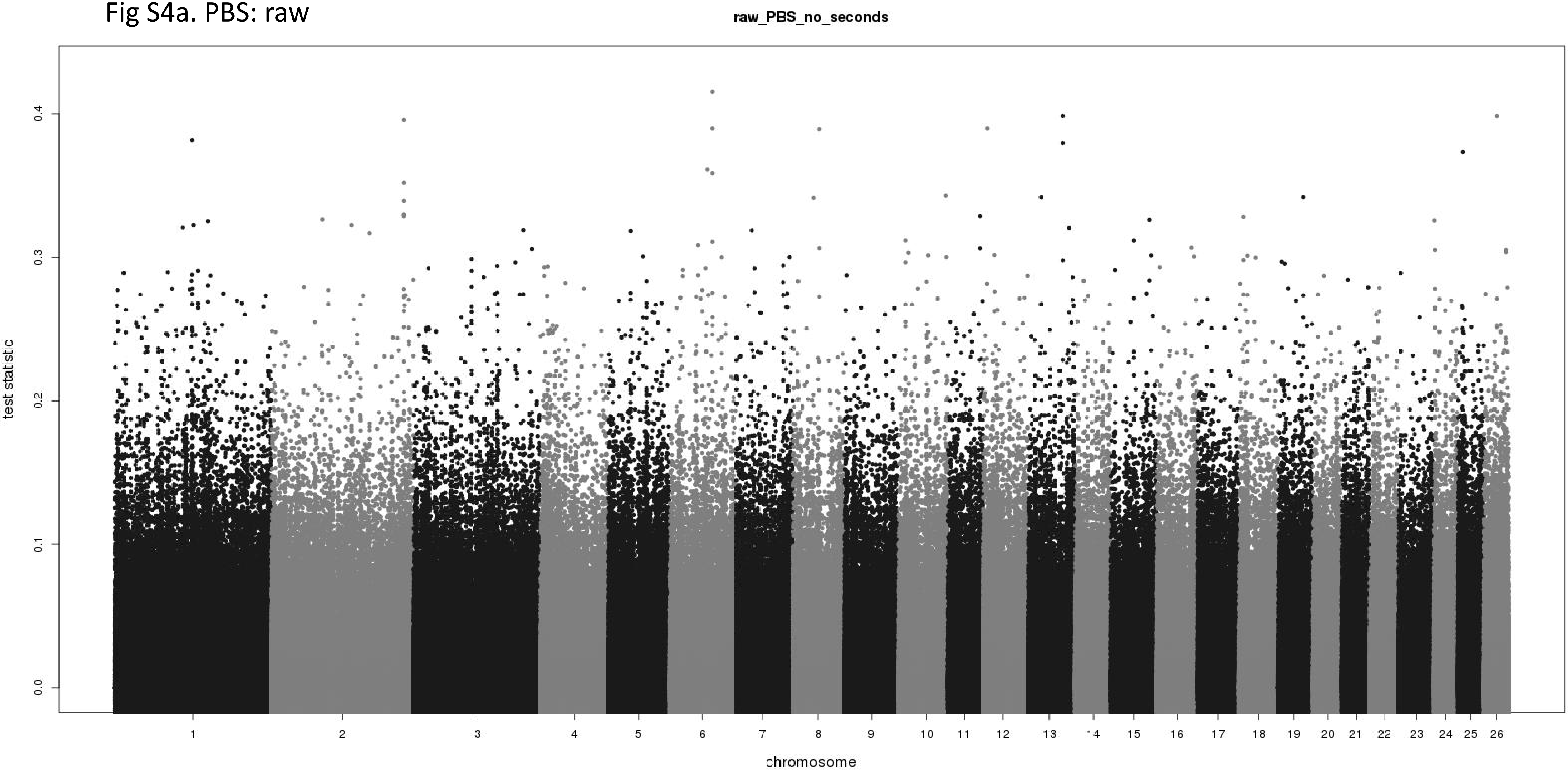

Fig S4b. PBS: mean

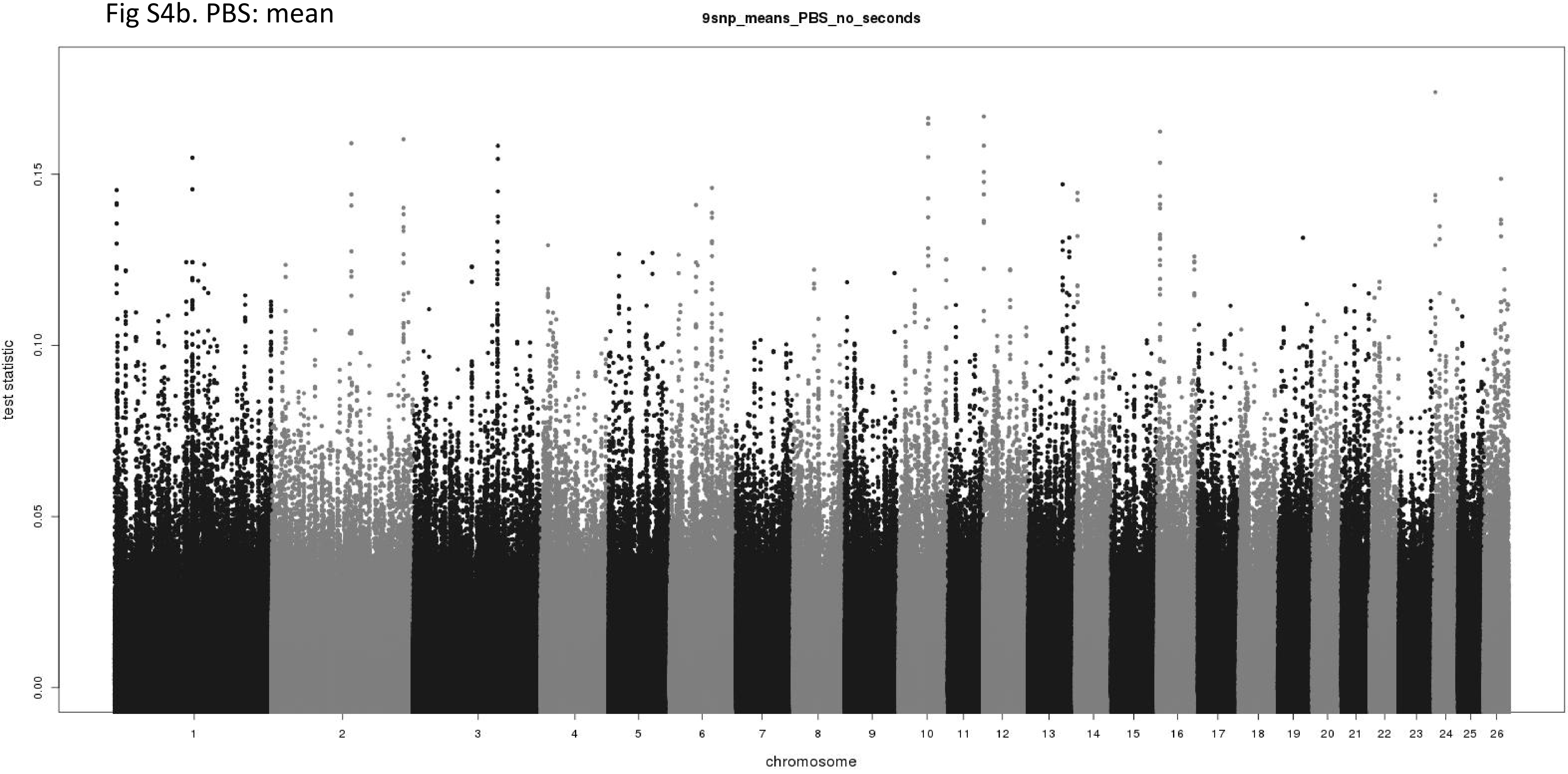

Fig S4c. PBS: median

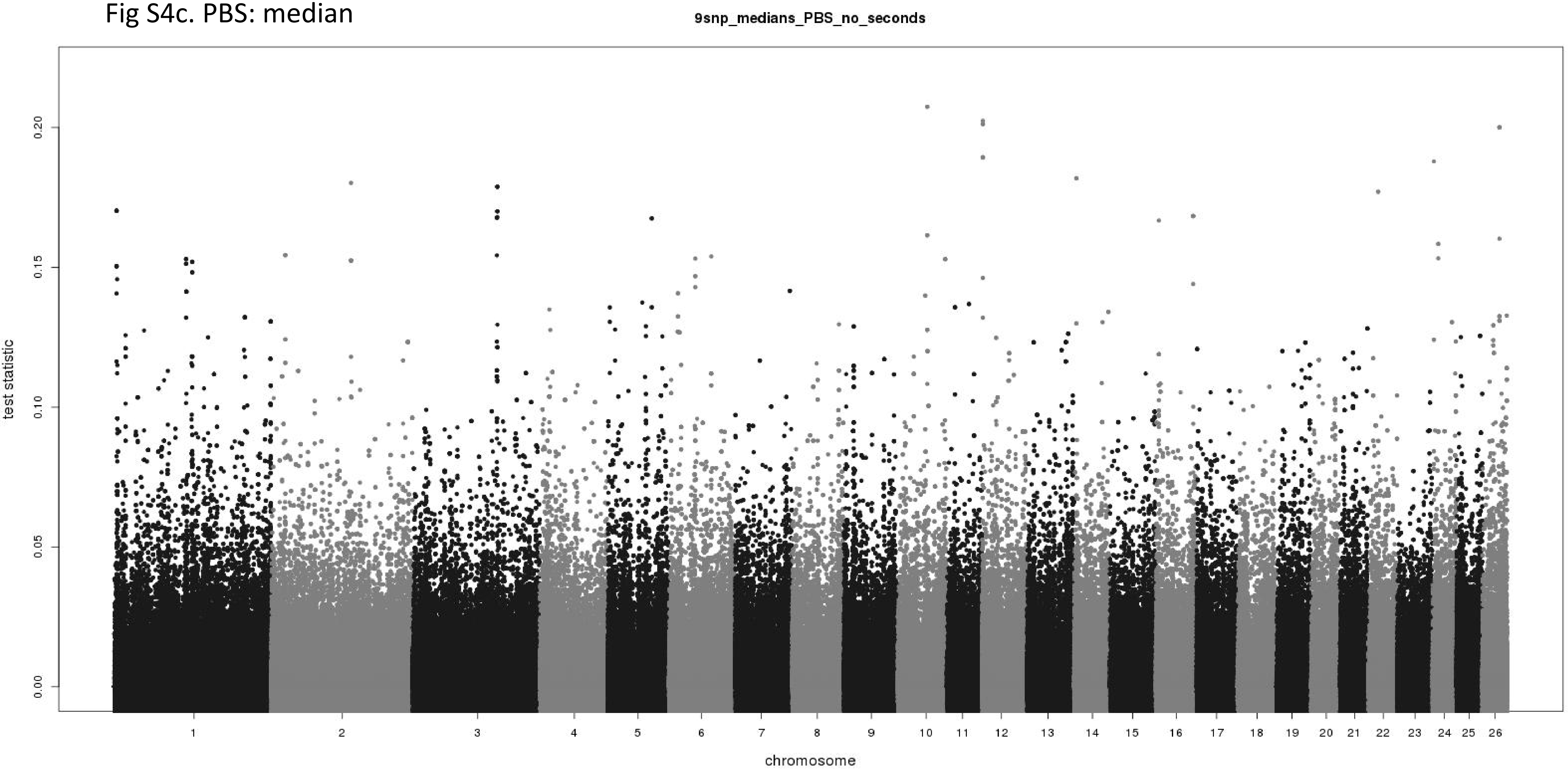

Fig S5a. env1: raw

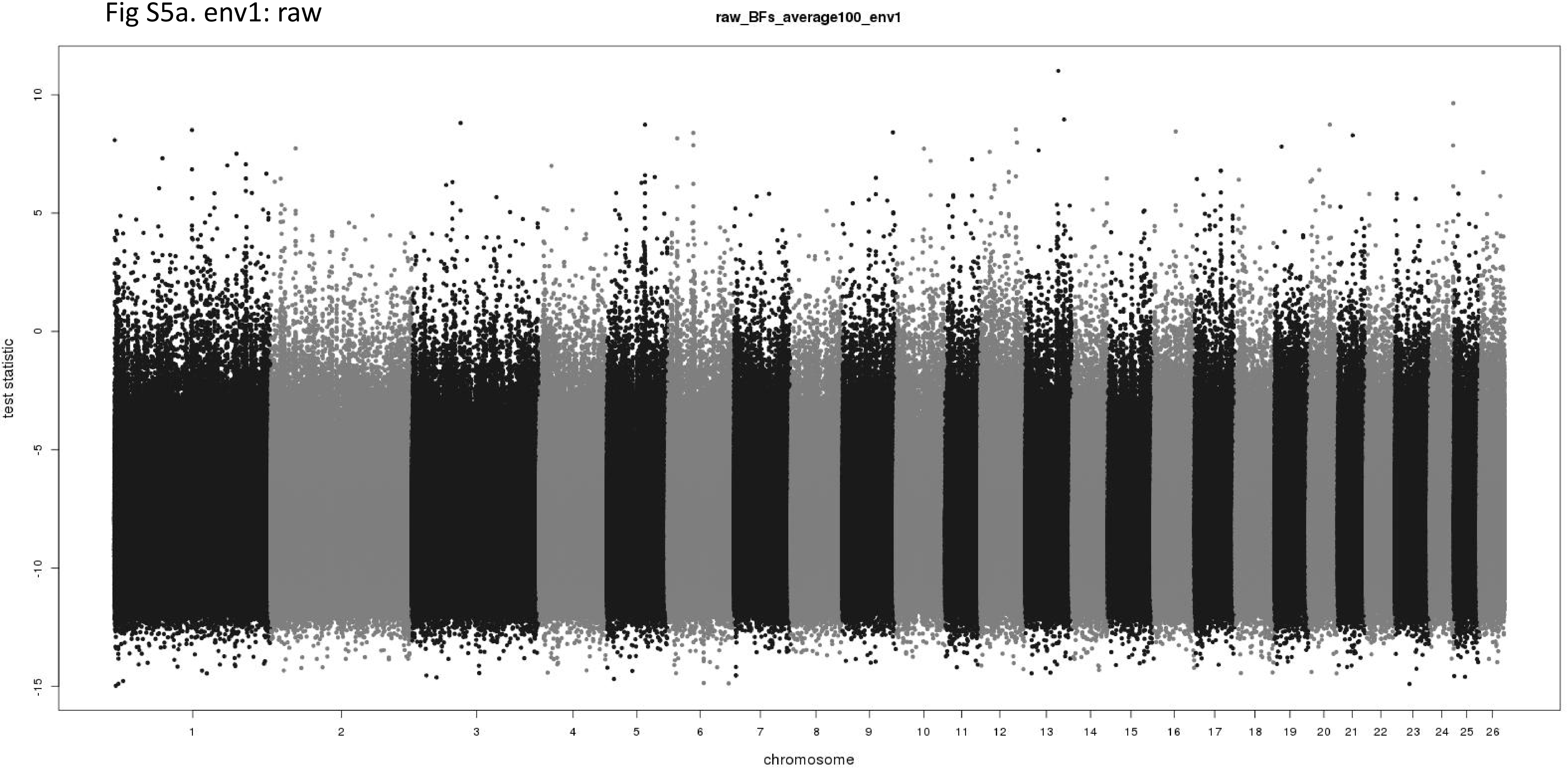

Fig S5b. env1: mean

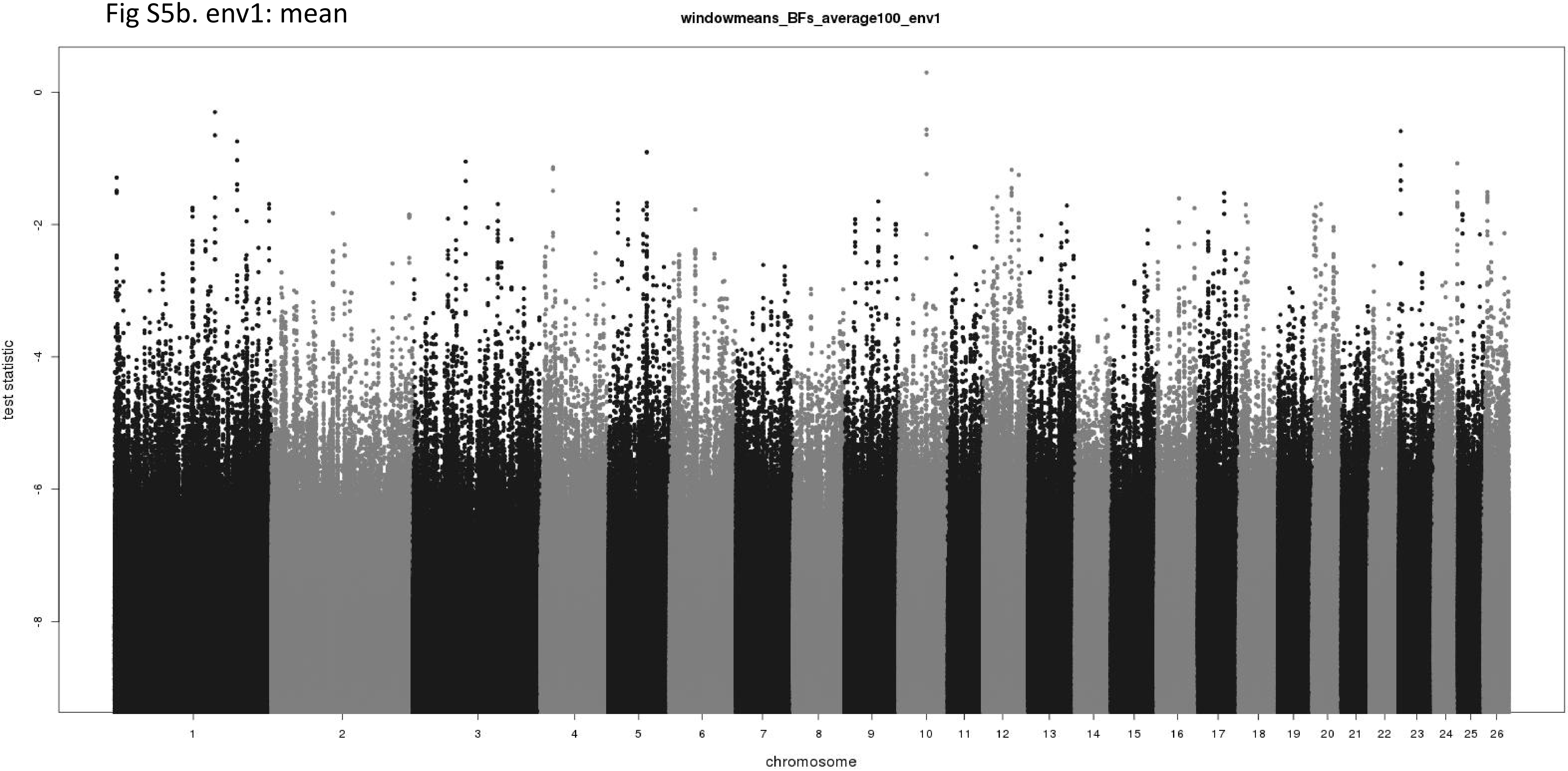

Fig S5c. env1: median

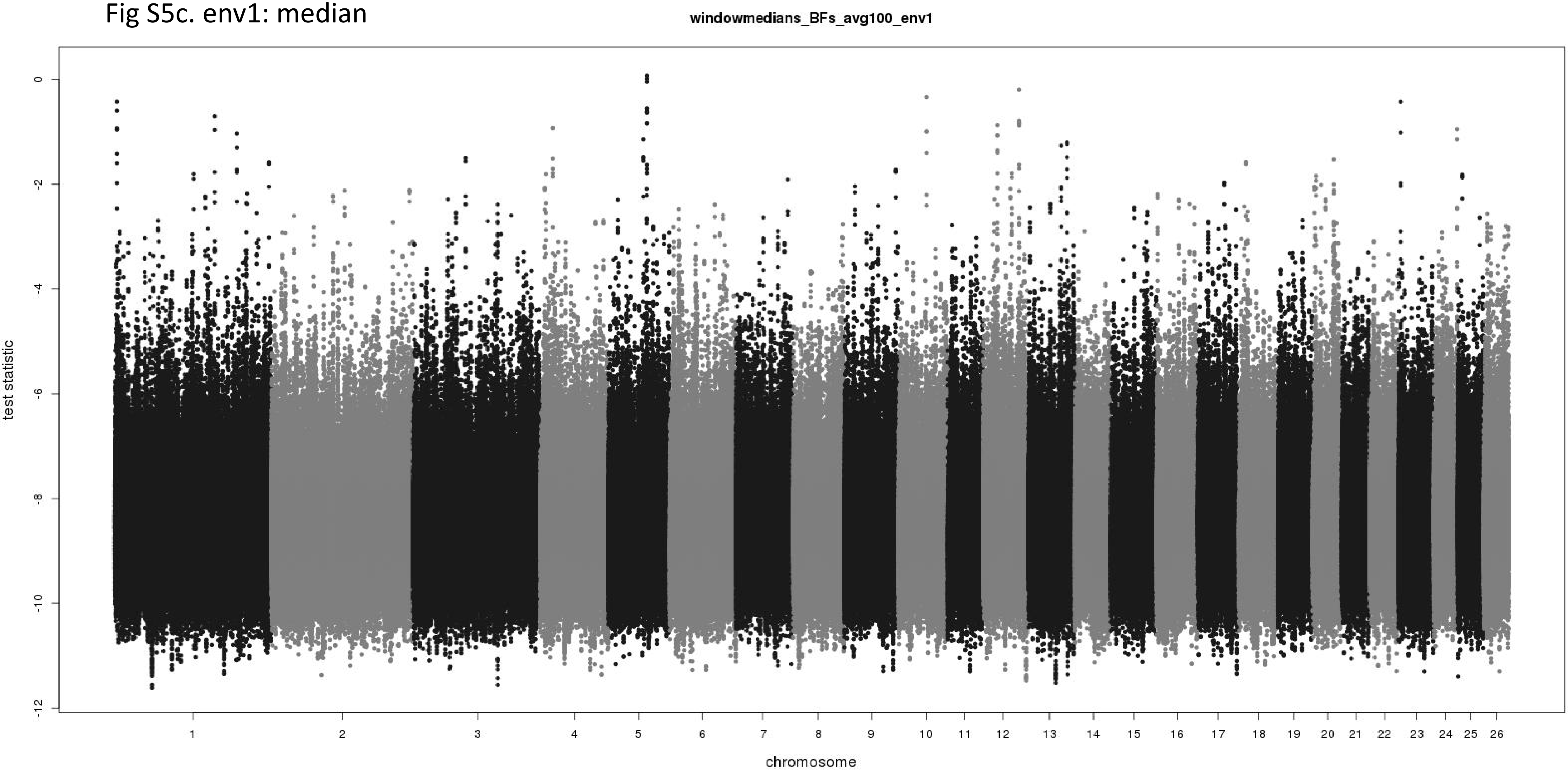

Fig S6a. env3: raw

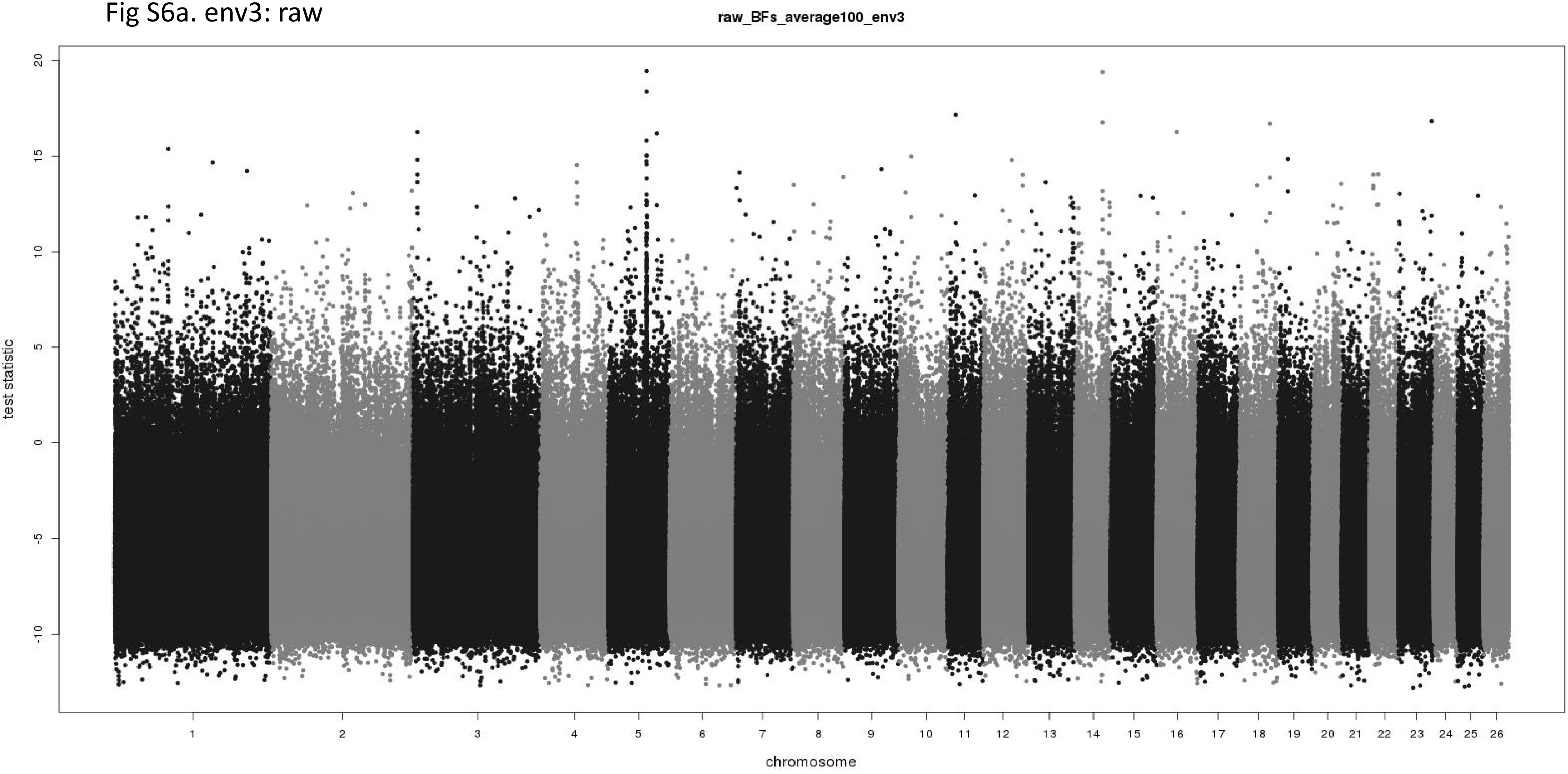

Fig S6b. env3: mean

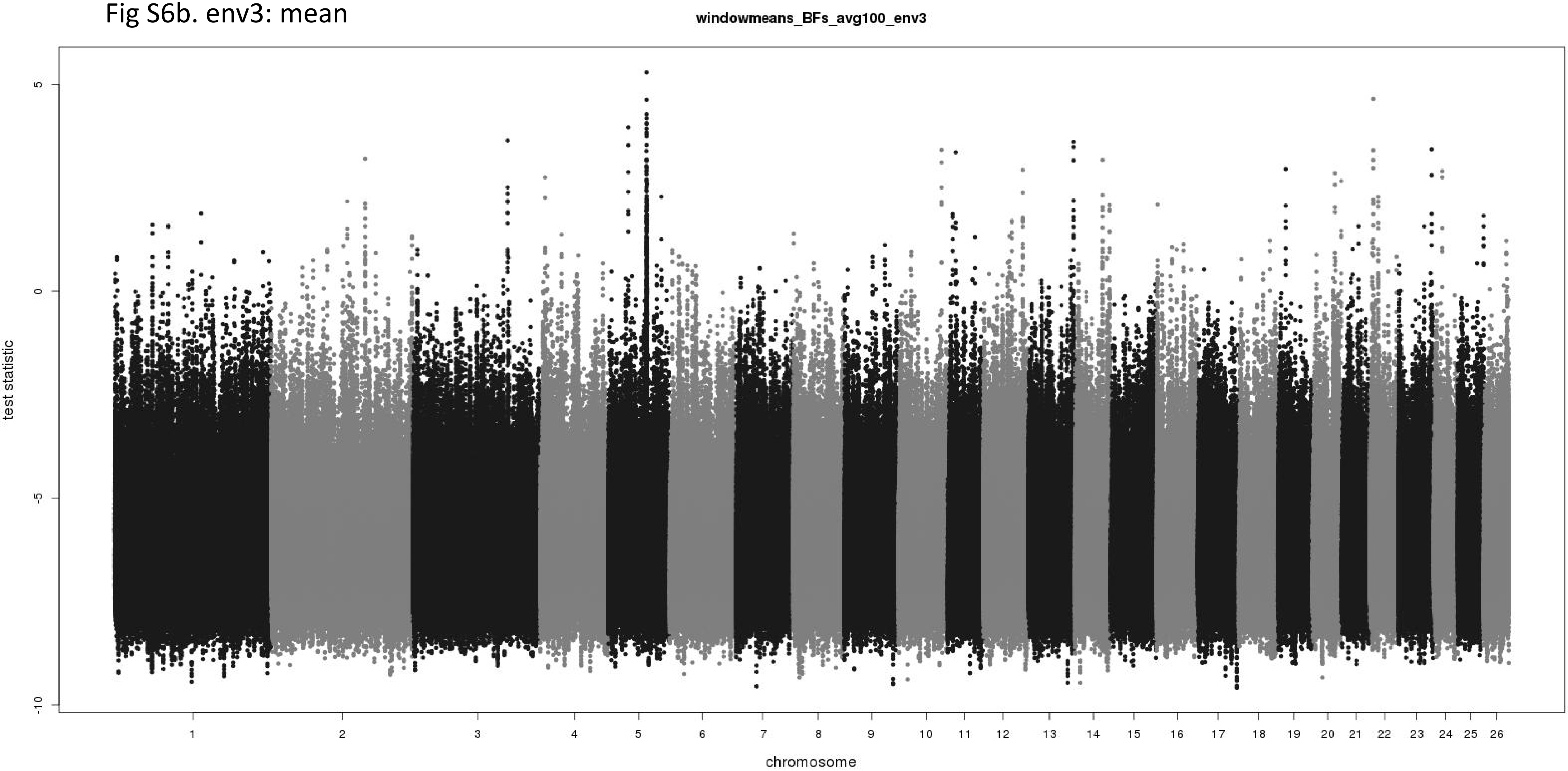

Fig S6c. env3: median

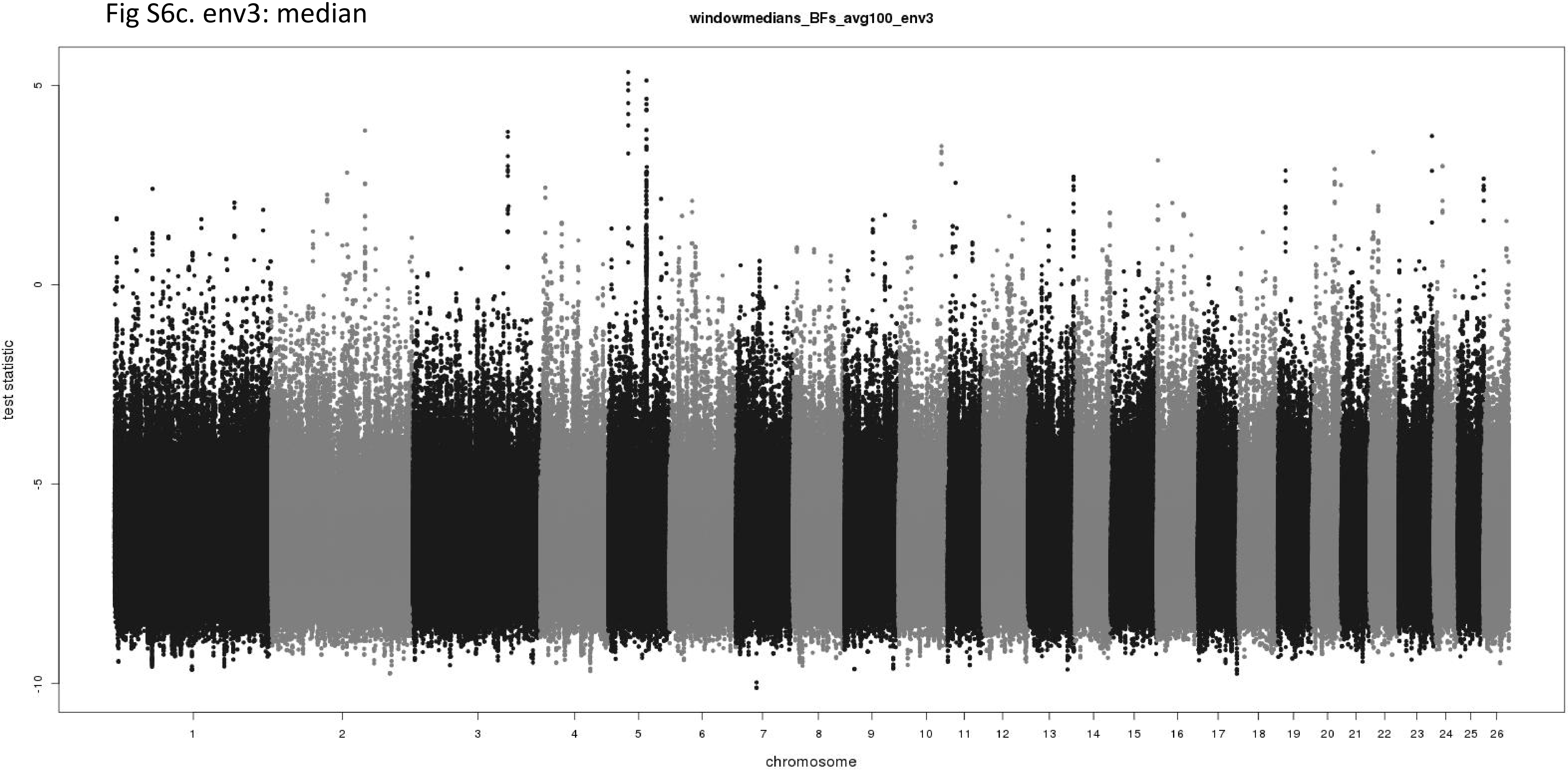

Fig S7a. env6: raw

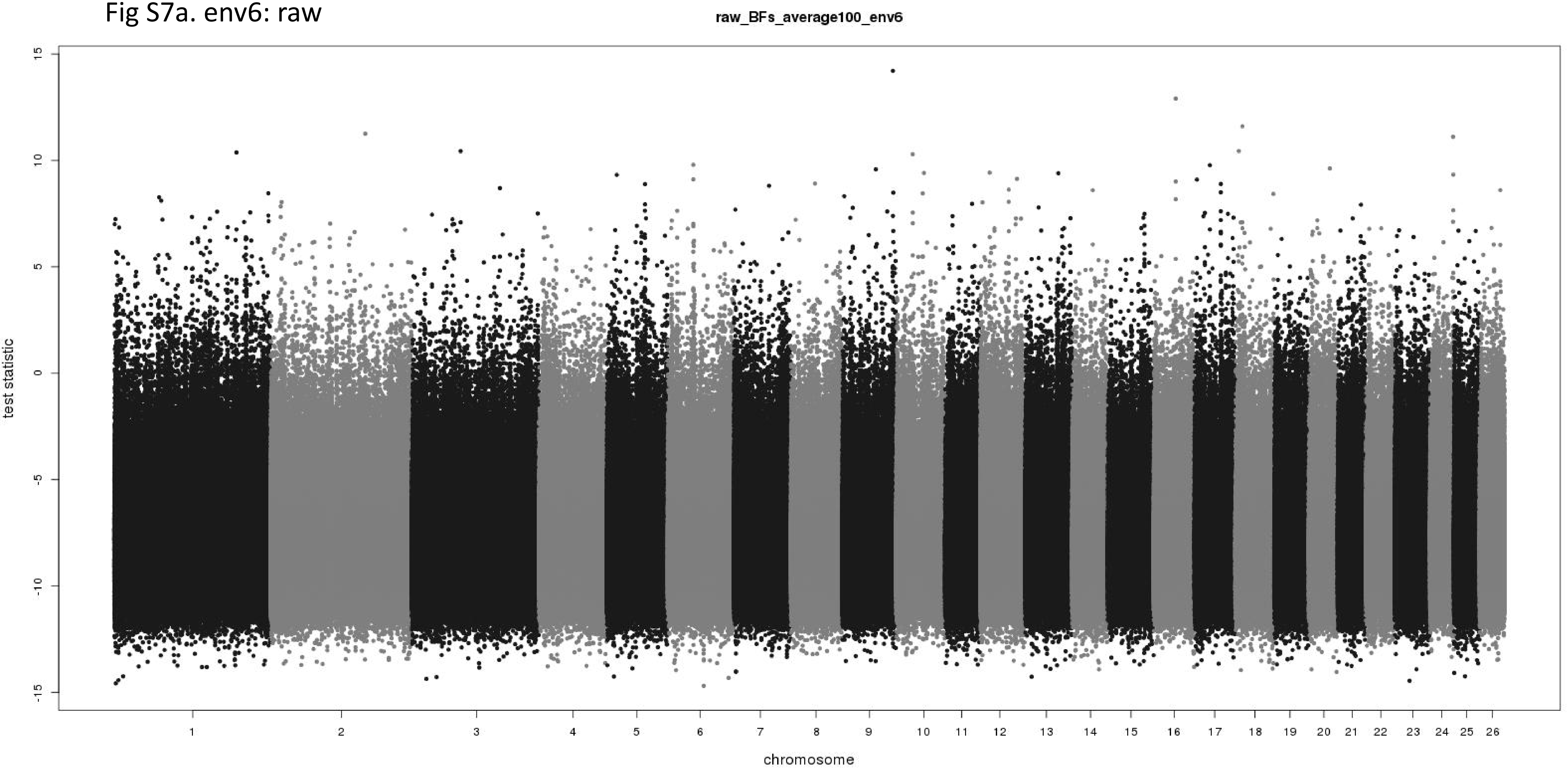

Fig S7b. env6: mean

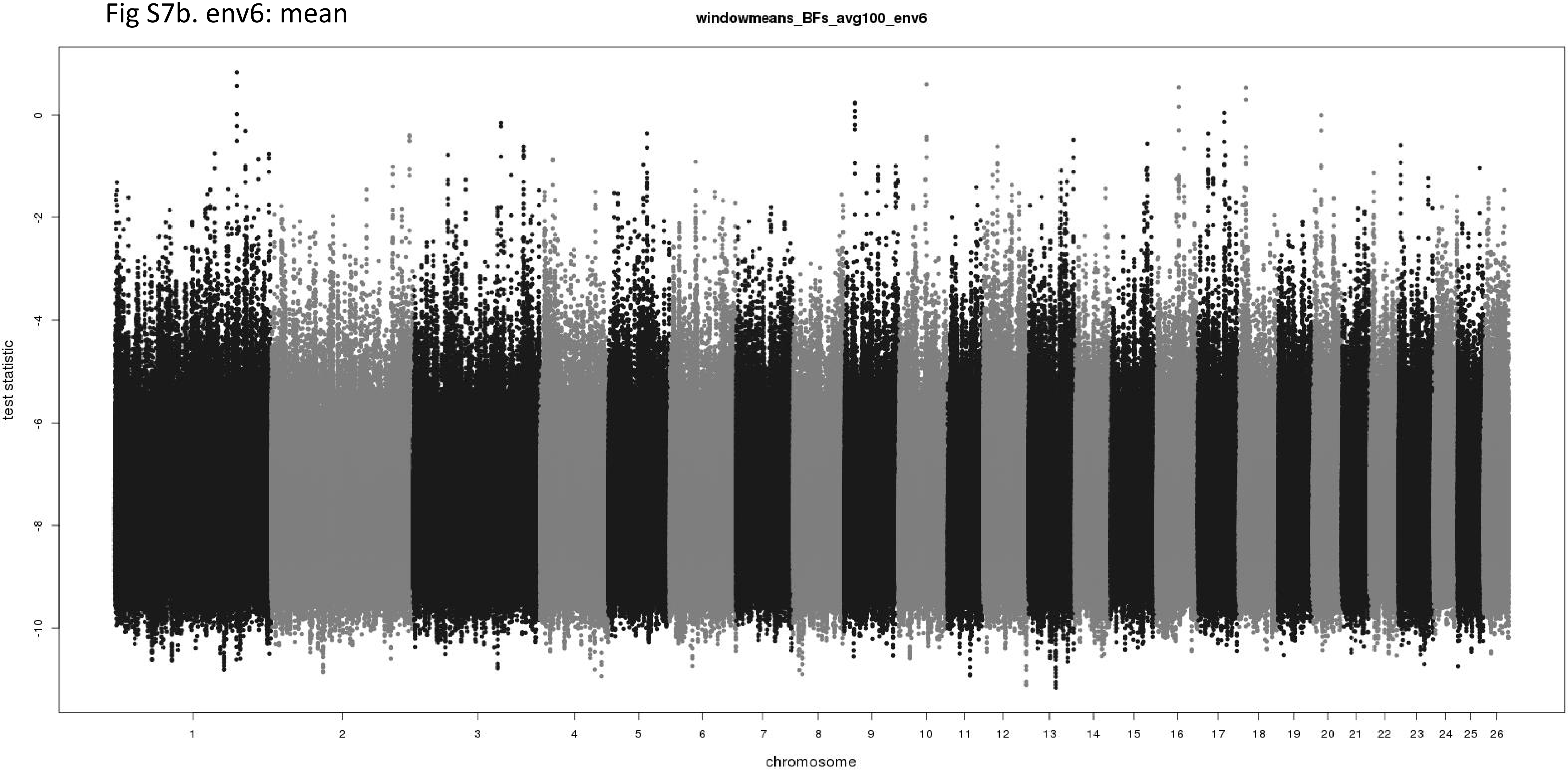

Fig S7c. env6: median

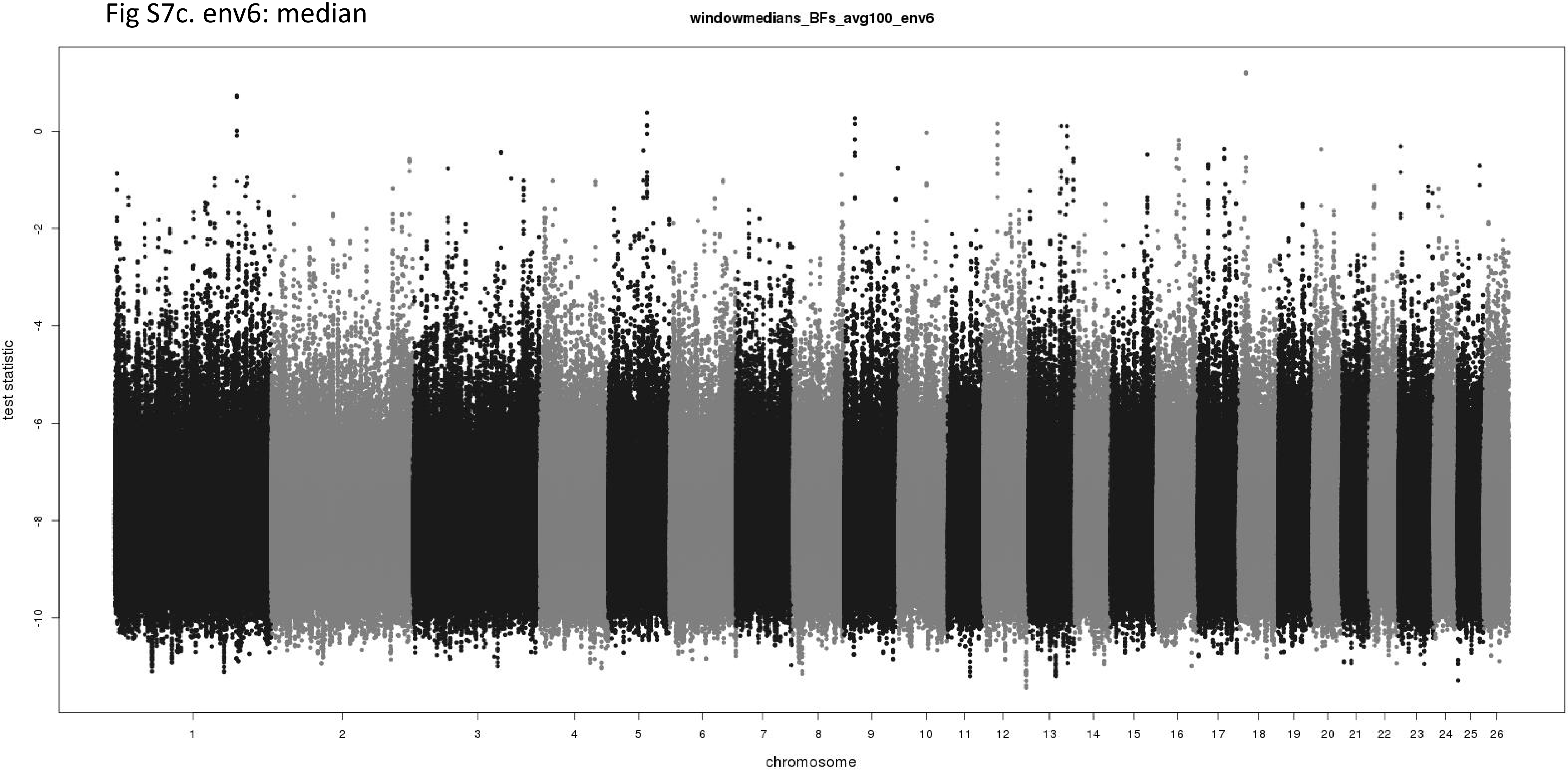

Fig S8a. env13: raw

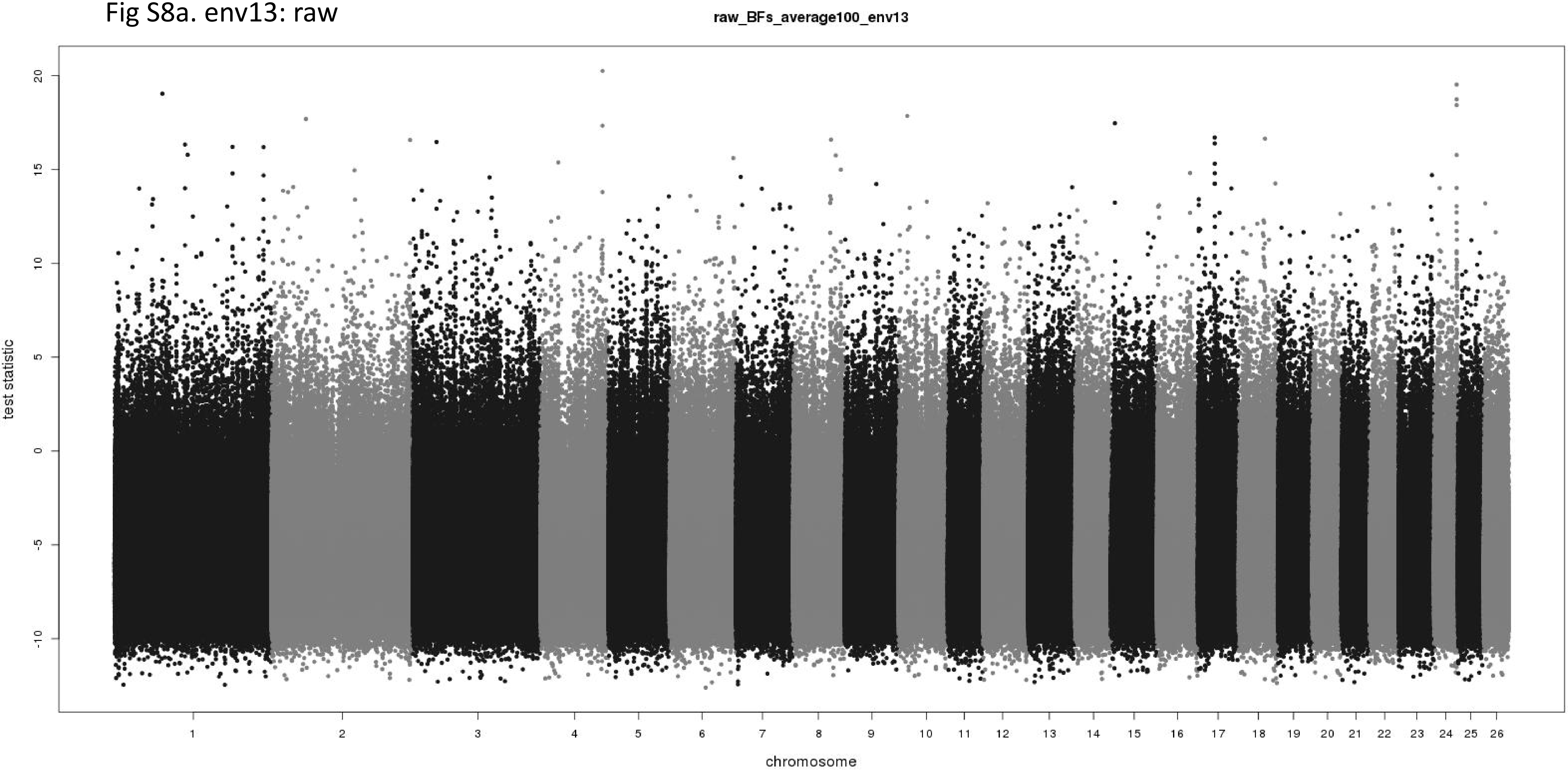

Fig S8b. env13: mean

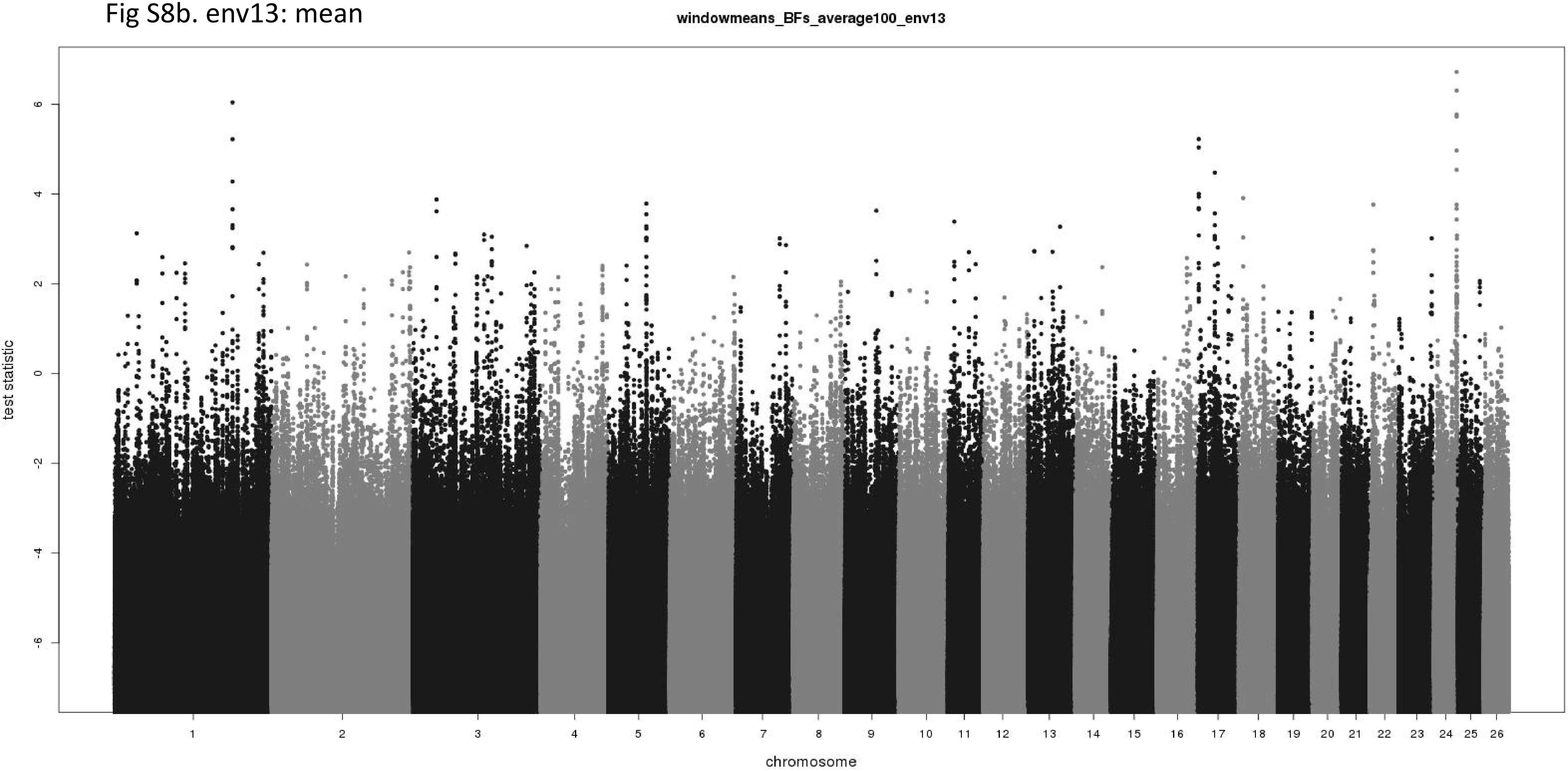

Fig S8c. env13: median

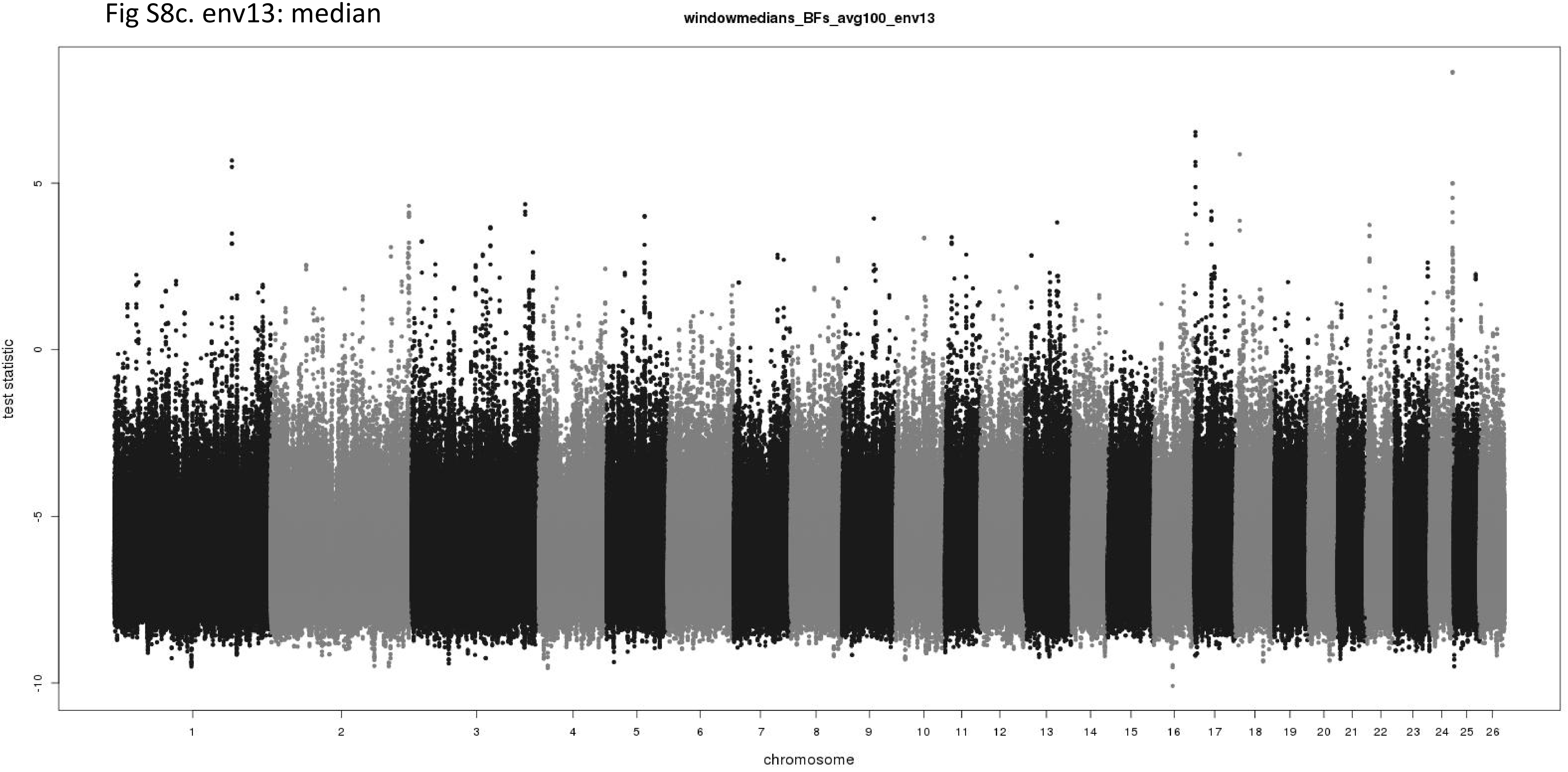

Fig S9a. env17: raw

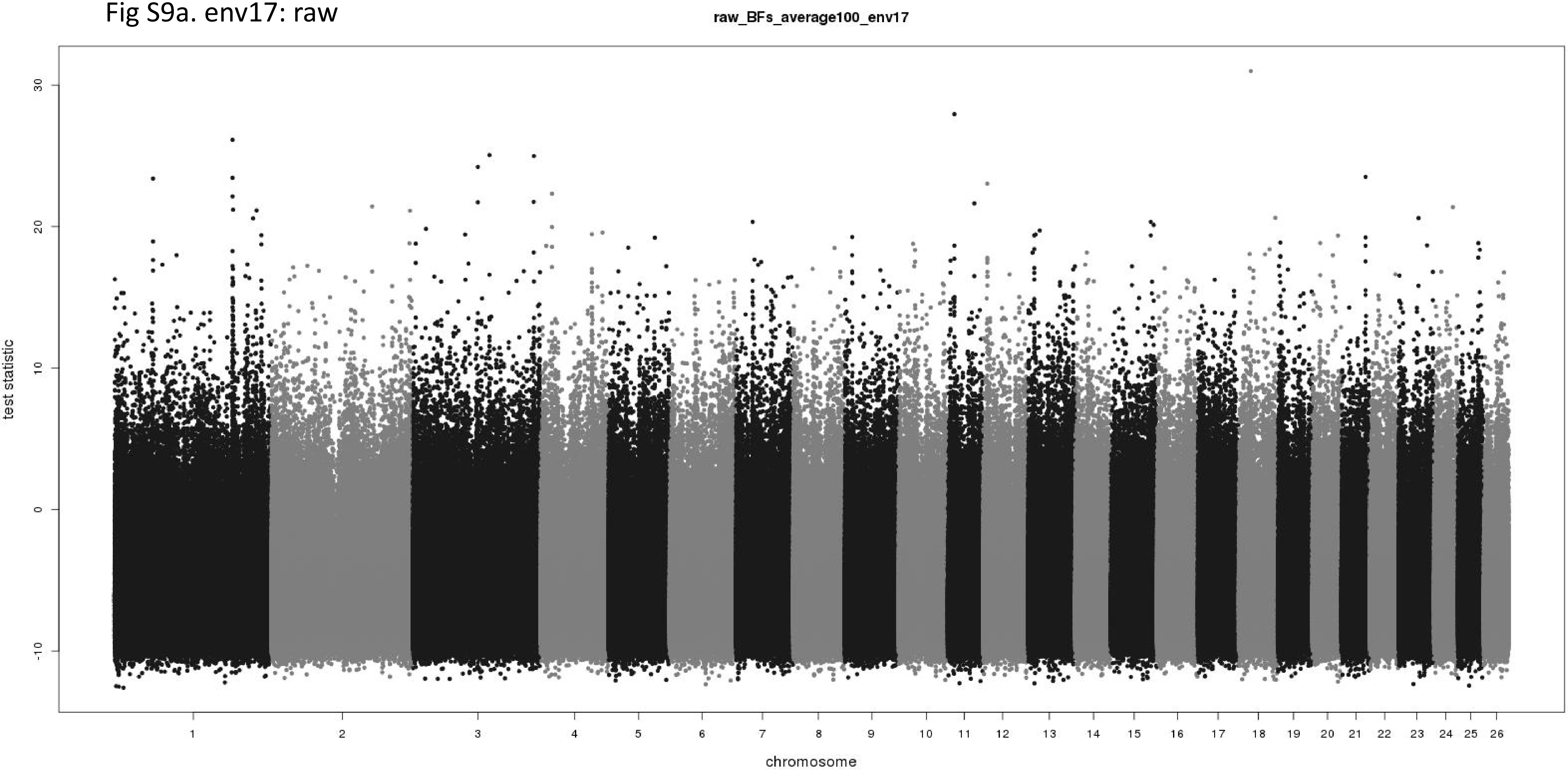

Fig S9b. env17: mean

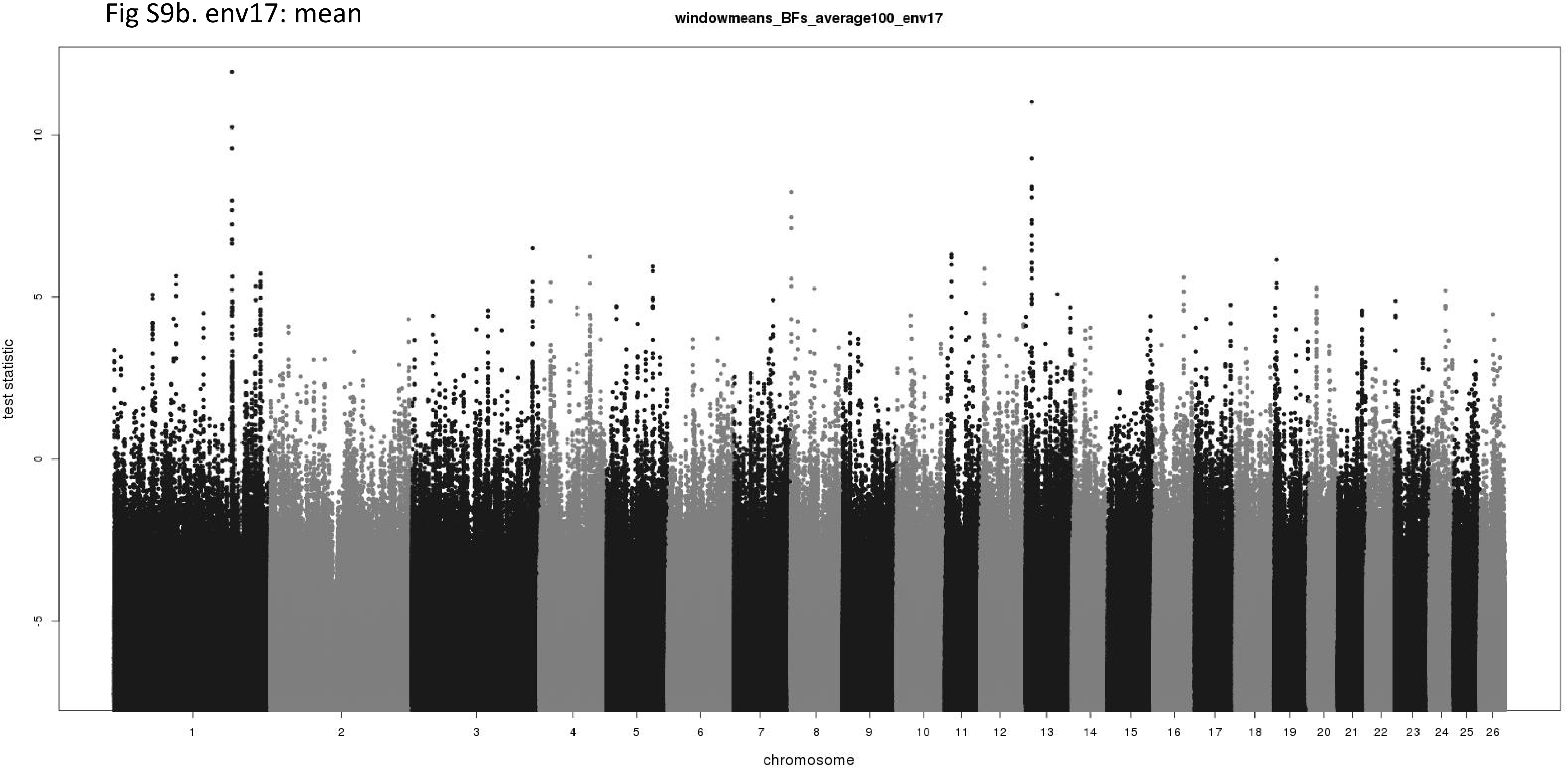

Fig S9c. env17: median

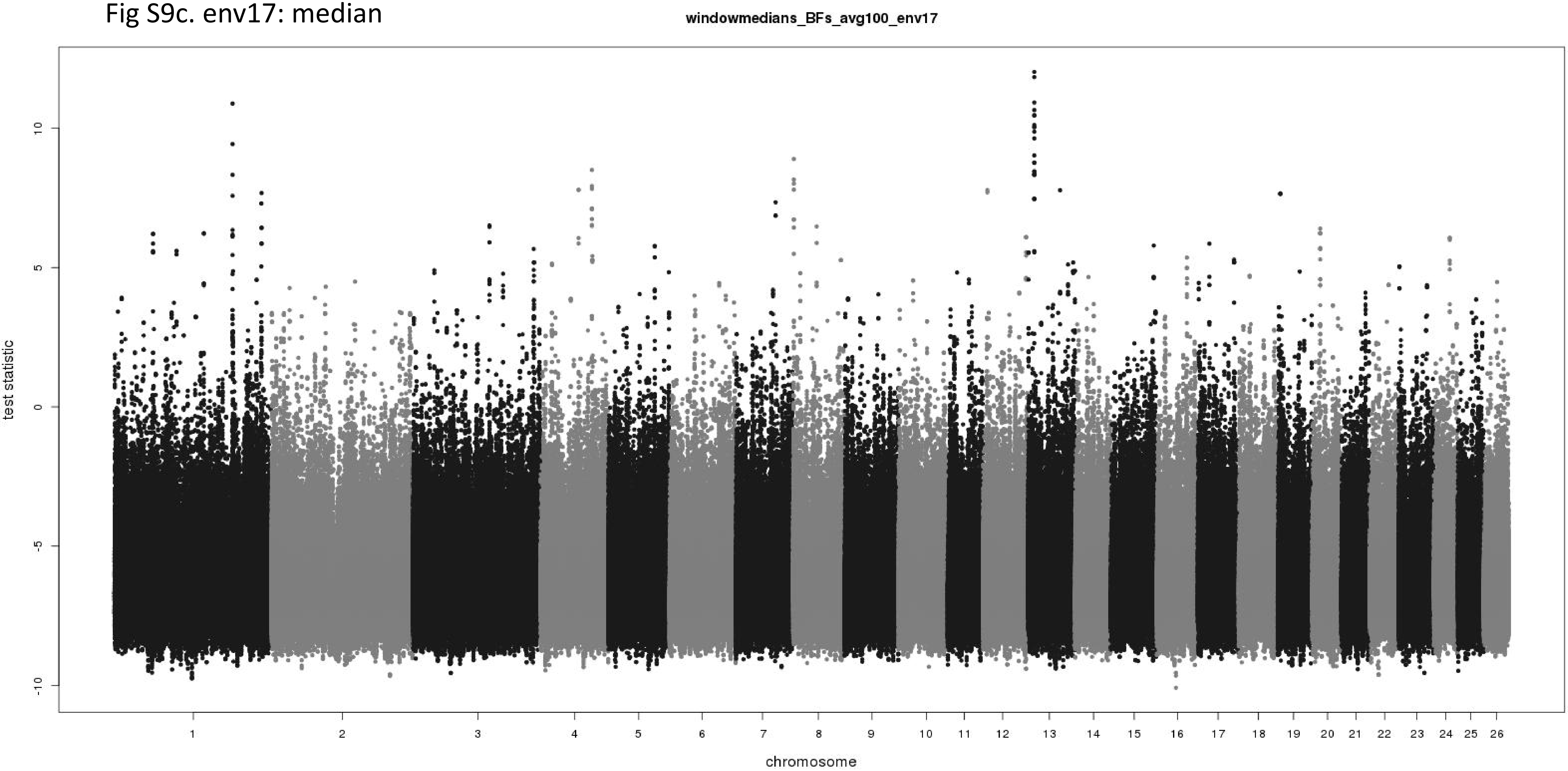

Supplement: evab014_Supplementary_Data [file evab014_supplementary_data.zip › Supp_tablesS1_S5-8_figuresS1-9.pdf]
